# A compendium of developmental gene expression in Lake Malawi cichlid fishes

**DOI:** 10.1186/s12861-017-0146-0

**Published:** 2017-02-03

**Authors:** R. F. Bloomquist, T. E. Fowler, J. B. Sylvester, R. J. Miro, J. T. Streelman

**Affiliations:** 1 Georgia Institute of Technology, School of Biological Sciences and Institute for Bioengineering and Bioscience, Atlanta, GA USA; 2 Medical College of Georgia, School of Dentistry, Augusta, GA USA

**Keywords:** Cichlid fishes, Evolution of gene expression, Lake Malawi, Developmental pathways

## Abstract

**Background:**

Lake Malawi cichlids represent one of a growing number of vertebrate models used to uncover the genetic and developmental basis of trait diversity. Rapid evolutionary radiation has resulted in species that share similar genomes but differ markedly in phenotypes including brains and behavior, nuptial coloration and the craniofacial skeleton. Research has begun to identify the genes, as well as the molecular and developmental pathways that underlie trait divergence.

**Results:**

We assemble a compendium of gene expression for Lake Malawi cichlids, across pharyngula (the phylotypic stage) and larval stages of development, encompassing hundreds of gene transcripts. We chart patterns of expression in Bone morphogenetic protein (BMP), Fibroblast growth factor (FGF), Hedgehog (Hh), Notch and Wingless (Wnt) signaling pathways, as well as genes involved in neurogenesis, calcium and endocrine signaling, stem cell biology, and numerous homeobox (Hox) factors—in three planes using whole-mount *in situ* hybridization. Because of low sequence divergence across the Malawi cichlid assemblage, the probes we employ are broadly applicable in hundreds of species. We tabulate gene expression across general tissue domains, and highlight examples of unexpected expression patterns.

**Conclusions:**

On the heels of recently published genomes, this compendium of developmental gene expression in Lake Malawi cichlids provides a valuable resource for those interested in the relationship between evolution and development.

## Background

Comparative gene expression is a hallmark of the evolution and development research program [1]. This is particularly the case among closely related vertebrate species, like hominids [2], beach mice [3], cavefishes [4], stickleback [5], and cichlid fishes [6]. In these examples and many others, diversity in key traits evolves via spatial, temporal and/or quantitative variation in gene expression. Despite the importance of changes in gene expression to the evolution of closely related species, comprehensive surveys of spatial expression patterns are typically confined to laboratory models (e.g., zebrafish, [7]).

**Fig. 1:**
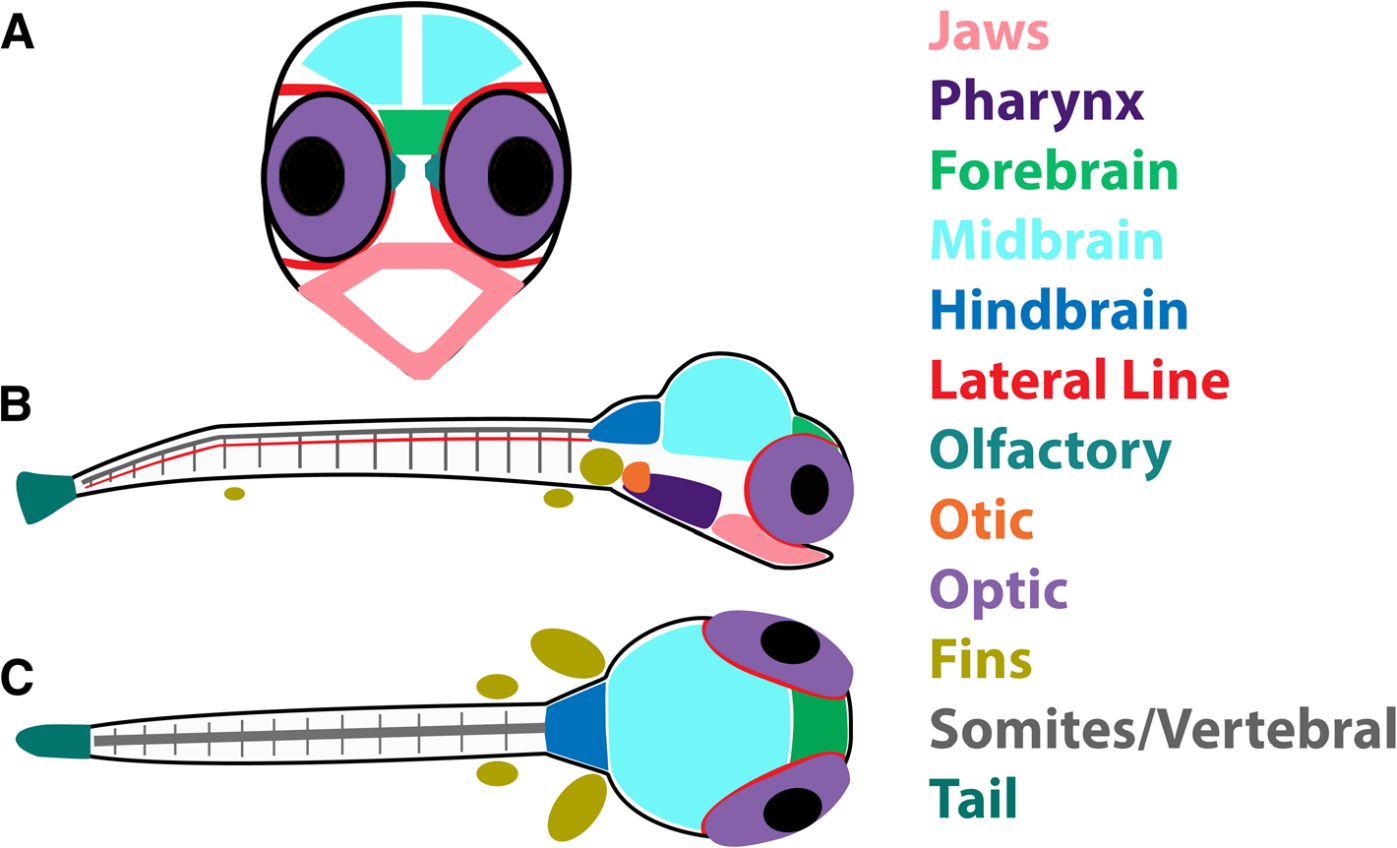
Schematic of planes: **a** frontal, **b** lateral, **c** dorsal planes and domains of expression color coded according to legend

Consequently, we have produced a compendium of spatial gene expression across early development, in Lake Malawi cichlid fishes. This resource should find broad applicability for three reasons. First, genomic surveys demonstrate extreme genetic similarity among Malawi species [8, 9], and other lineages from East Africa [10]. This means the probes we develop will be useful across hundreds of African cichlid species. Second, Malawi cichlids in particular have been used to study the genetic and developmental basis of key vertebrate phenotypes, like nuptial coloration [11–13], the cranio-dental skeleton [14–18], and the brain [19]. The developmental pathways we highlight here are relevant to continued study of these important evolutionary phenotypes. Third, recently developed means to manipulate cichlid genomes and development (e.g., transgenics, treatment with small molecules, genome editing: [17, 20, 21] are informed by observations of gene expression in time and space. We use whole mount *in situ* hybridization (ISH) to document spatial expression patterns for approximately 160 genes, across 12 major categories. We tabulate expression domains for each gene at pharyngula and larval stages, in three planes of view (Fig. 1). This compendium of developmental gene expression should be a valuable resource for biologists interested in the relationship between development and evolution.

## Methods

### Fish husbandry

Lake Malawi cichlids used for this study included *Metriaclima zebra* [MZ] and *Petrotilapia chitimba “thickbar”* [PC]. These species were used owing to their availability and the fact that they belong to the ‘mbuna’ rock dwelling lineage. While Malawi cichlid species share qualitative expression domains across species, those from different ecotypes (mbuna versus ‘non-mbuna’) may exhibit heterochronic and quantitative differences in expression [6, 17]. Adult cichlids were maintained in re-circulating aquarium systems at 28 °C (Georgia Institute of Technology). Fertilized embryos were removed from the mouths of brooding females and staged in days post-fertilization (dpf), according to the Nile tilapia developmental series [22]. Embryos were raised to 4dpf or 6dpf and euthanized with sodium bicarbonate buffered anesthetic MS-222, before fixation in 4% paraformaldehyde. Pre-hatching embryos at 4dpf were dechorionated using fine forceps to achieve proper fixation and reagent penetration.

### Primer and probe design

Primers were designed using recently assembled and annotated tilapia and MZ genomes [10] (accession numbers KT906433-KT906561) as well as partial genome assemblies [9] (accession numbers KC633830- KC633846, EU867210-EU867217, KT851376-- KT851399) were used to amplify cichlid cDNA. Amplified DNA was inserted into the pGEM-T Easy vector system (Promega) and transformed into JM109 competent cells (Promega). Upon amplification and purification (Qiagen, Plasmid Maxi Kit), riboprobes were prepared using the Promega Riboprobe System Sp6/T7 kit. There is minimal sequence divergence between Malawi cichlid species; the average nucleotide diversity is 0.28%, less than that among laboratory strains of zebrafish [9]. Cloned plasmid insert sequences used for probe generation have been deposited in GenBank (Accession numbers are shown in Tables 1, 2, 3, 4, 5, 6, 7, 8, 9, 10, 11).

**Table 1:**
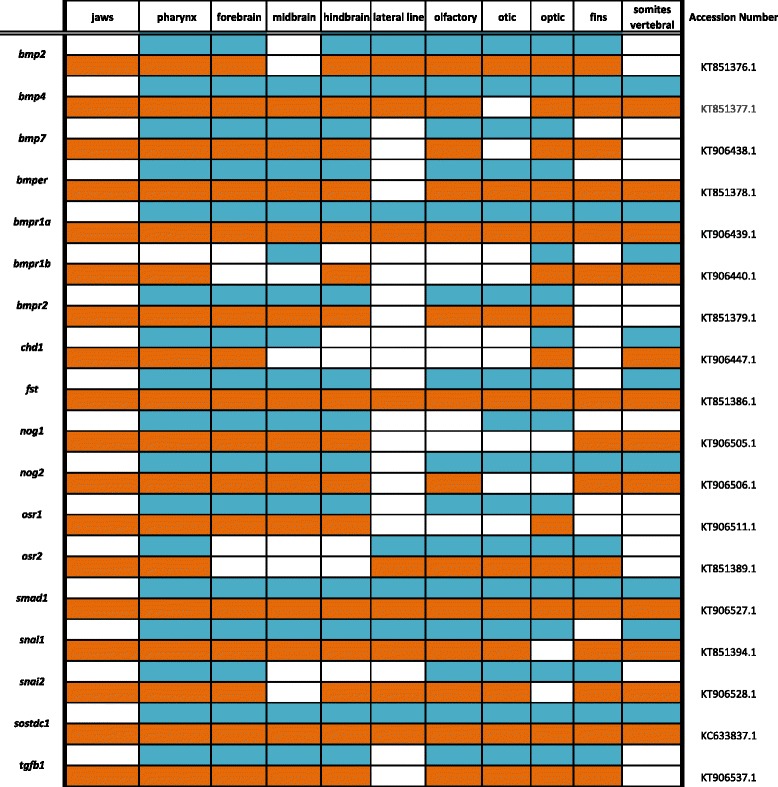
Expression data for the TGF-β/BMP pathway genes at pharyngeal (blue) and larval (orange) stages

**Table 2:**
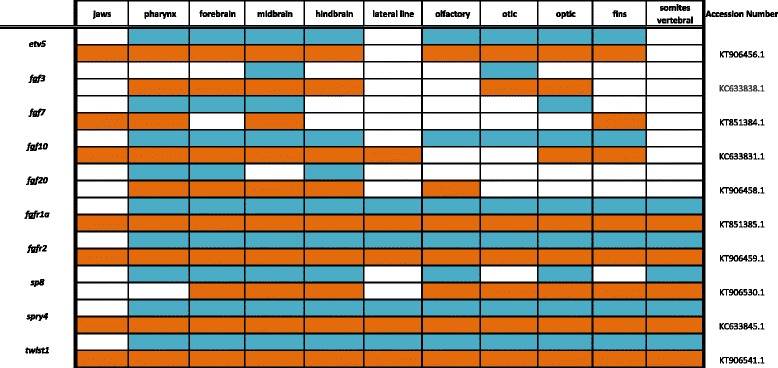
Expression data for FGF pathway genes at pharyngeal (blue) and larval (orange) stages

**Table 3:**
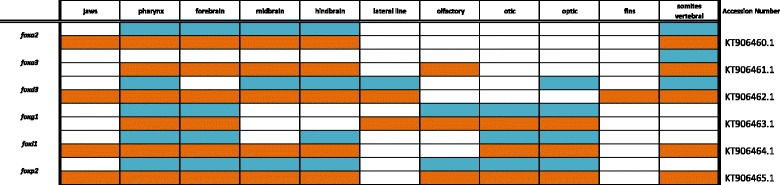
Expression data for Forkhead Box family genes at pharyngeal (blue) and larval (orange) stages

**Table 4:**
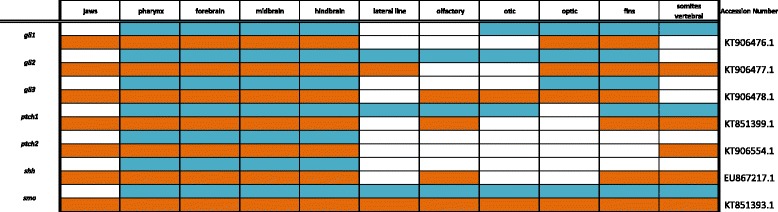
Expression data for Hedgehog pathway genes at pharyngeal (blue) and larval (orange) stages

**Table 5:**
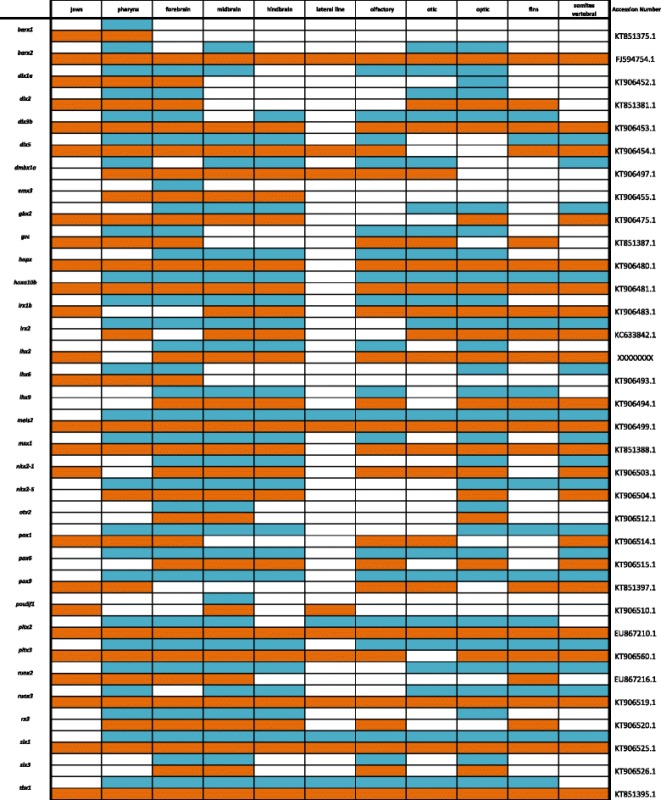
Expression data for Homeobox pathway genes at pharyngeal (blue) and larval (orange) stages

**Table 6:**
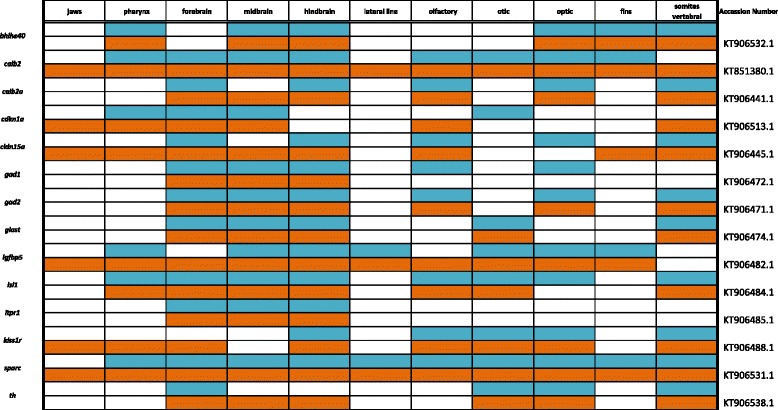
Expression data for Calcium, Endocrine, and Insulin signaling factors at pharyngeal (blue) and larval (orange) stages

**Table 7:**
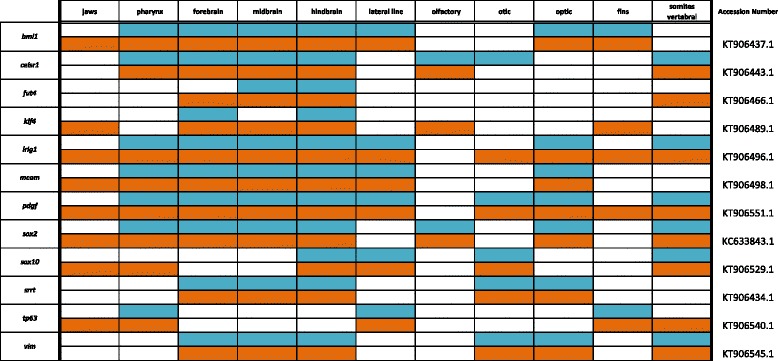
Expression data for Mitogens, Stem, and Tumor Suppressor factors at pharyngeal (blue) and larval (orange) stages

**Table 8:**
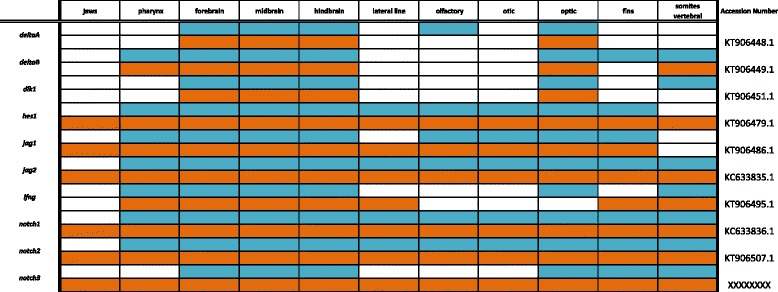
Expression data for Notch pathway genes at pharyngeal (blue) and larval (orange) stages

**Table 9:**
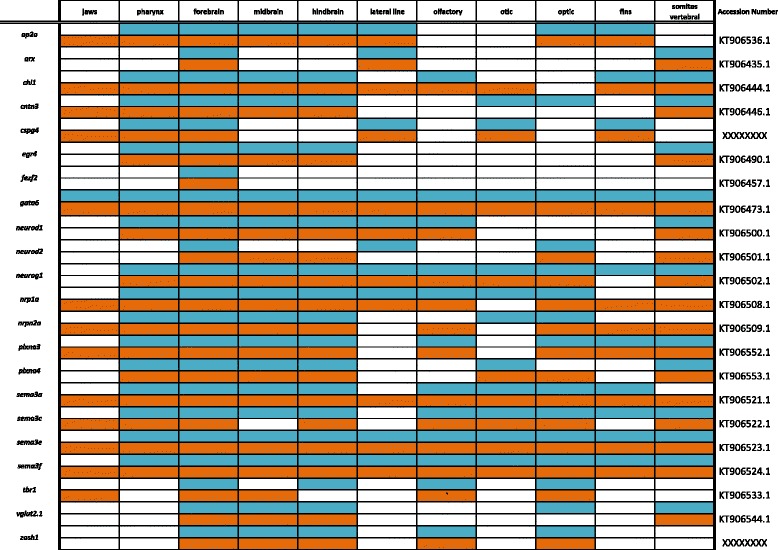
Expression data for brain development and neurogenesis factors at pharyngeal (blue) and larval (orange) stages

**Table 10:**
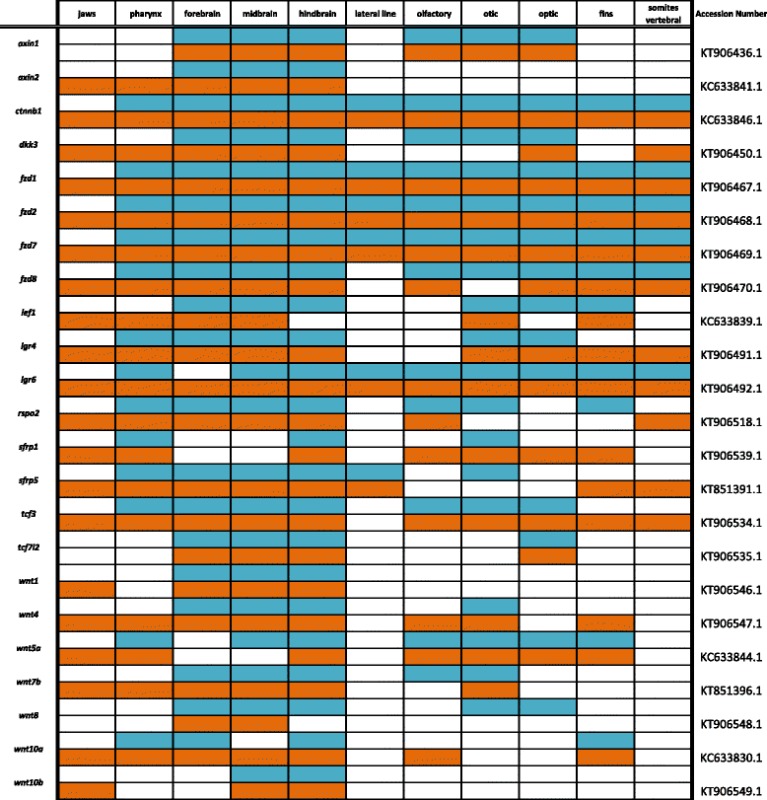
Expression data for brain development and neurogenesis factors at pharyngeal (blue) and larval (orange) stages

**Table 11:**
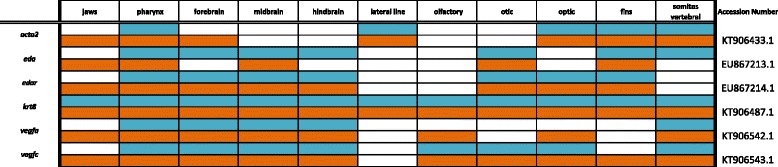
Expression data for developmental genes at pharyngeal (blue) and larval (orange) stages

### *In situ* hybridization

Specimens for ISH were fixed a minimum of 48 h in 4% paraformaldehyde at 4 °C and then dehydrated into a graded series of methanol for further fixation and storage at −20 °C O/N. Embryos were rehydrated and permeabilized in 10 μg/mL proteinase K for one hour. They were then refixed in 4% PFA and incubated in prehybridization solution at 70 °C. Embryos were incubated overnight at 70 °C in digoxigenin-labeled antisense riboprobes. The following day, embryos were washed through a graded series of saline-sodium citrate buffer solutions and blocked with blocking solution (5% blocking buffer, 5% goat serum in MABT). Embryos were then hybridized with 1:3000 anti-digoxigenin-AP FAB fragments in blocking buffer overnight at 4 °C. Excess antibody was removed by washing, and color reaction with NBT/BCIP was performed on the AP-conjugated anti-dig antibodies. Gene expression was imaged in whole mount, using a LeicaDFC295 compound light microscope.

## Results and discussion

### Bone morphogenetic protein and transforming growth factor beta pathway

The transforming growth factor beta (TGF-β) superfamily is a class of cytokines organized into TGF-βs, bone morphogenetic proteins (BMPs), and activin/inhibins that bind to Type I and II serine/threonine kinase receptors [23]. Upon ligand activation, type II receptors phosphorylate type I receptors, leading to SMAD protein activation and ultimately gene regulation. TGF-β/BMPs play a major role in almost every aspect of vertebrate biology, from gastrulation and organization of the body plan, to the genesis of almost every organ, to renewal and adult tissue maintenance [23, 24]. Inasmuch, mutations in the TGF-β superfamily and its regulators have been demonstrated as causative for the evolution of major adaptations.

BMPs are believed to control multiple aspects of cichlid jaw shape and function [25, 26], are in part responsible for evolutionary novelty in beak shape of Darwin’s finches [27], and have a direct dose-dependent effect on the craniofacial skeleton when transgenically titrated in mice [28]. All of the BMP pathway factors we include are expressed in the jaw once it has formed in the larval stage, and many are also expressed in the pharynx, as indicated in Table 1. *bmp2* and *bmp4* pattern, generate, shape, and regenerate teeth in mice, squamates, and cichlids [29–35] while a large-effect QTL containing *bmp6* has been reported for a doubling of pharyngeal tooth number in stickleback [36]. *bmper* may regulate tooth number in Malawi cichlids [17].

**Fig. 2:**
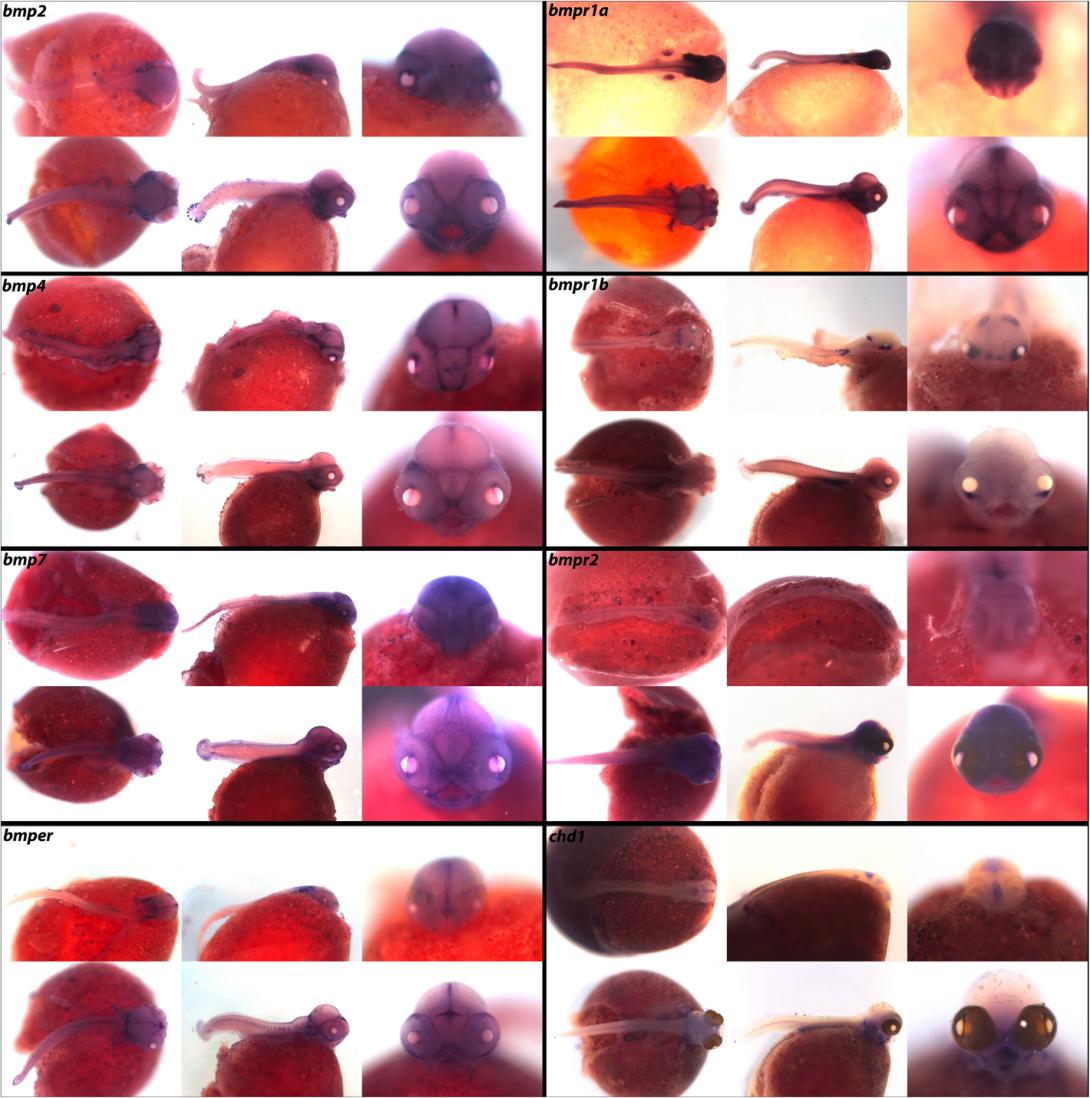
Expression of genes from the TGF-β/BMP pathway at pharyngeal and larval stages. Imaged whole-mount in dorsal, lateral, and frontal orientations

In mice, *bmp2* and *bmp7* regulate dorso-ventral patterning of the brain and in chicken, undifferentiated neural ectoderm has been induced to express dorsal-specific markers by addition of the protein products of these genes [37]. In Fig. 2 we observe expression of ligands *bmp2*, *bmp4* and *bmp7,* as well as endothelial regulator *bmper* and receptors *bmpr1a* and *bmpr2,* along the dorsomedial telencephalon and in distinct patterns in the forebrain, similar to expression reported in mouse [38]. After mutation of *bmpr1a* in mouse the choroid plexus fails to form from the dorsal telencephalon, demonstrating a role of this receptor in forebrain specification [39]. While *bmpr1a* is expressed in all three regions of the brain, *bmpr1b* is localized to the developing cerebellum, preoptic region, and eyes at 4dpf; and at 6dpf is seen in the eyes and somites. *chd1* is expressed in the optic nerve, pharynx, notochord, and gut.

**Fig. 3:**
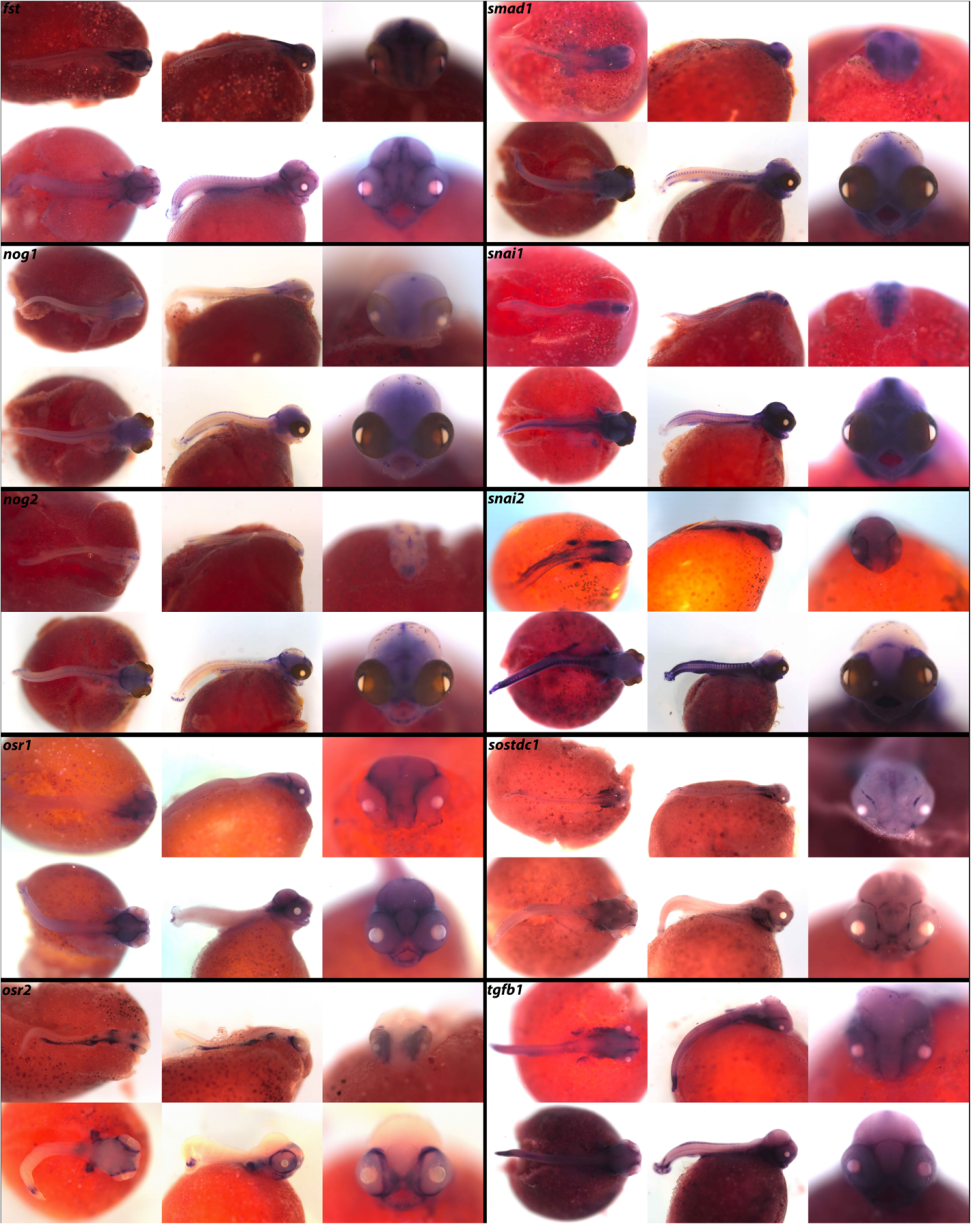
Expression of genes from the TGF-β/BMP pathway at pharyngeal and larval stages. Imaged whole-mount in dorsal, lateral, and frontal orientations

In Fig. 3, BMP inhibitor *fst* is heavily expressed throughout the brain at 4dpf, and by 6dpf this expression sharpens. Inhibitors *nog1* and *nog2* are seen in the vertebrae and brain, with *nog1* diffusely throughout the brain and exhibiting localized expression in the hindbrain and somites at 6dpf. *nog2* demonstrates more restricted areas of expression in the preoptic region and lens at 4dpf, and additional expression in the developing dental placodes at 6dpf. Transcription factor *osr1* is expressed in the optic tectum and hindbrain at both stages, and *osr2* is heavily expressed in the gut and behind the eyes at 4dpf and in the retinae at 6dpf.

Tumor-suppressor *smad1* is phosphorylated in response to BMP pathway activation, and regulates transcription. We observe *smad1* throughout the brain, fins, eyes, somites/vertebrae, jaw, and pharynx. Transcription factors *snai1* and *snai2* exhibit distinct expression patterns. *snai1* is in all three brain regions, notably along the longitudinal fissure at 6dpf, as well as the vertebrae/somites. *snai2* appears around the eyes pretectum, and pectoral fins, along with heavy expression in the pharyngeal arches and somites. We observe *sostdc1* in the pharynx and cranial lateral line, and *tgfb1* around the eyes, retinae, pharynx, and fins.

### Fibroblast growth factor pathway

Much like the TGF-β/BMP pathway, the Fibroblast growth factor (FGF) pathway plays a part in eukaryotic development and homeostasis across ontogeny, and is particularly important for organogenesis and the generation of evolutionary novelty. FGFs and FGF receptors (FGFRs) are part of a larger family of Tyrosine Kinases and high-affinity cell surface receptors known as Receptor-Tyrosine Kinases (RTKs) that function through activation of Ras/MAP kinase and phospholipase-C gamma pathways [40]. Conserved across all metazoans, FGFs have gained redundancy in higher vertebrate genomes, presumably for the formation of complex traits. In Amphioxus, FGF’s coordinate segment reduction, perhaps permissive for the evolution of the vertebrate head [41]. In frog, FGFs work synergistically with BMPs to induce neurulation [42]. By contrast, FGFs have long been recognized as competitors of the BMP pathway in patterning limb outgrowth in mammals [43], and formation and regeneration of fish fins [44]. Genetic ablation of the FGF antagonists *Spry4* and *Spry2* produces mice with tusks for incisors [45], and heterochrony of *fgf8* expression in the blind cavefish neural plate contributes to defective retinal morphogenesis [4]. It is apparent that FGFs are one of the key pathways exploited by nature during animal evolution [46].

**Fig. 4:**
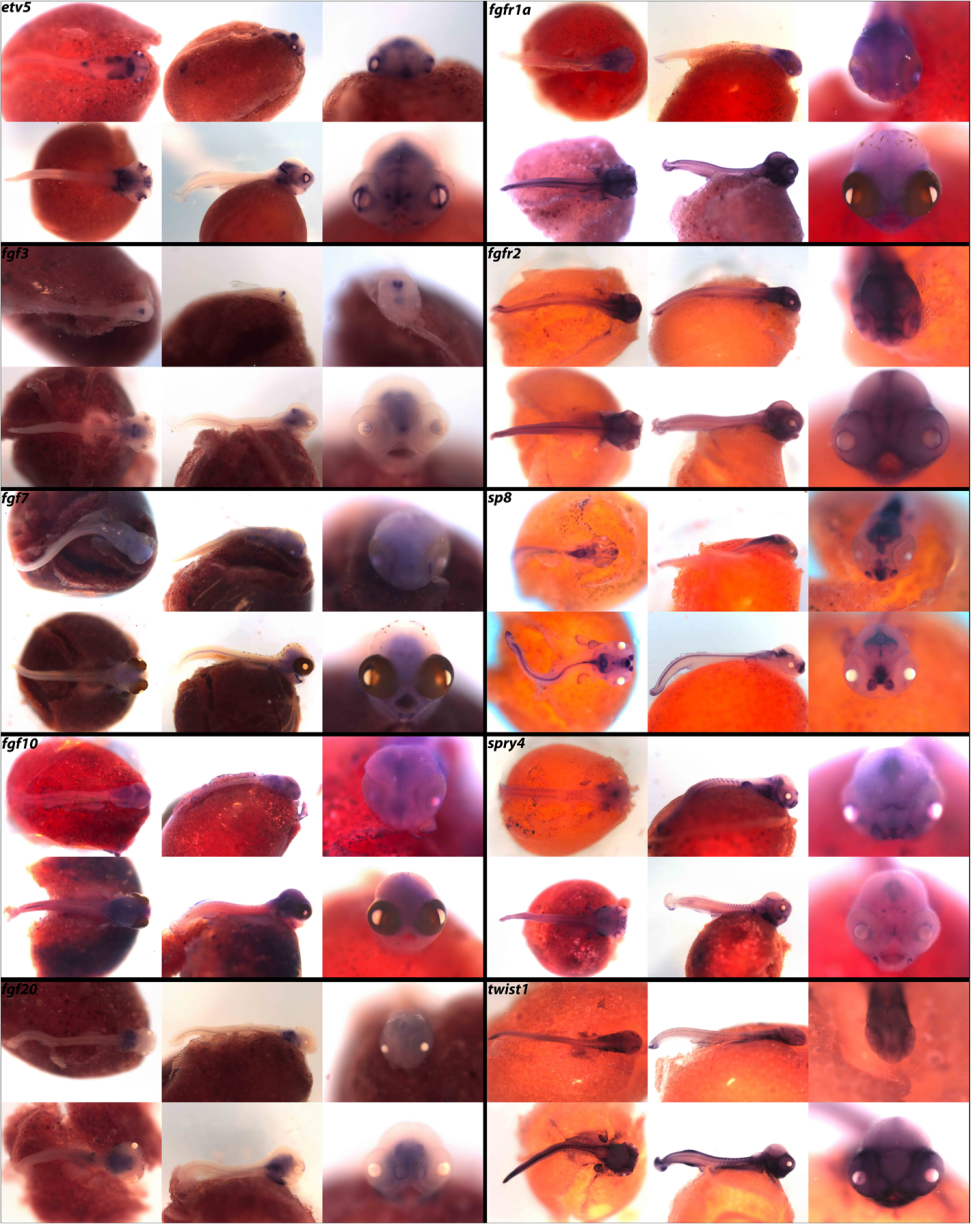
Expression of genes from the FGF pathway at pharyngeal and larval stages. Imaged whole-mount in dorsal, lateral, and frontal orientations

In Fig. 4 and Table 2, we report expression of ligands *fgf3*, *fgf7*, *fgf10* and *fgf20*; receptors *fgfr1a* and *fgfr2*; transcription factors *etv5, sp8* and *twist1*; and repressor *spry4*. Expression of *etv5* can be seen in the hindbrain, eyes, jaws, fins and pharynx. The four ligands included have unique expression patterns with *fgf3* most evident in the isthmus (midbrain-hindbrain boundary, MHB), *fgf7* in the jaw, *fgf10* in the midbrain and around the eyes, and *fgf20* in the hindbrain, olfactory placodes, and pharynx. Receptors *fgfr1a* and *fgfr2* are both heavily expressed in the central nervous system, along the longitudinal fissure of the brain, and in the pharynx and jaw.

Zinc-finger transcription factor *sp8* acts under the regulation of *fgf10* and Wnt/β-catenin, and has been shown to regulate *fgf8* for limb formation during chicken development [47]. We observe *sp8* along the spinal region and throughout the brain and olfactory placodes in a pattern similar to that seen in zebrafish [48]. Repressor *spry4* and transcription factor *twist1* are both expressed along the somites, as well as in the pharynx, fins, eyes, and jaws.

### Forkhead box pathway

The Forkhead Box transcription factors share an evolutionary conserved “forkhead” or “winged-helix” 100 amino-acid DNA binding domain. The moniker for the Fox family was coined when the first homolog *forkhead (fkh)* was identified in Drosophila, with mutant flies exhibiting split heads [49]. Moreover, the helix-turn-helix motif of this domain is comprised of 3 α-helices and two large loops that resemble “wings.” To date, over 100 Fox transcription factors have been identified across eukaryotes and much work has been done to clarify their nomenclature [50]. For example, in humans there are over fifty Fox proteins categorized into 19 subgroups (FOXA to FOXS) [51].

**Fig. 5:**
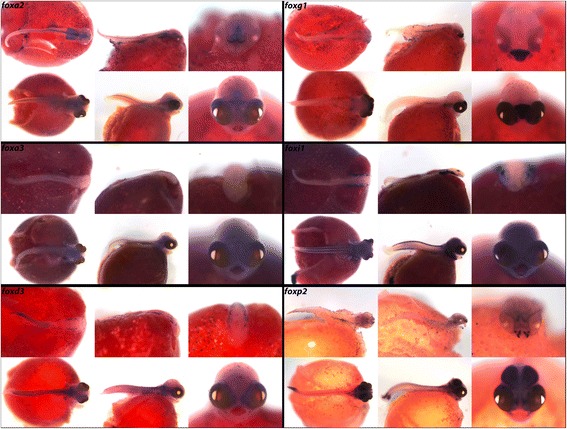
Expression of Forkhead Box genes at pharyngeal and larval stages. Imaged whole-mount in dorsal, lateral, and frontal orientations

While the characteristic protein domain of the Fox family has remained tightly conserved, individual genes have evolved greatly outside of these domains and have taken on a myriad of highly divergent and specialized functions such as tumor suppression, cell signaling, apoptosis, and DNA repair. Although functionally divergent, redundant roles exist for family members such as *FoxA1* and *FoxA2* in both lung and liver formation [52, 53]. In Fig. 5, we observe expression of *foxa2* in the diencephalon-midbrain boundary (DMB), the oral/pharyngeal cavity and in the developing spinal cord, while *foxa3* is expressed in the gut. *foxg1* is expressed in the retina and telencephalon, and is differentially expressed in rock- vs. sand-dwelling Malawi cichlids [6]. As in zebrafish, *foxi1* is expressed in pharynx, jaw, vertebral elements, and otic placodes [54] and in Malawi cichlids expression strongly resembles that of neural crest marker *foxd3*.

In vertebrates, FoxP2 has demonstrated roles in vocalization and the ability to learn language. Deletions in *FoxP2* result in verbal dyspraxia and a collapse of the communication system at both the neural and muscular levels [55]; furthermore Foxp2 has evolved episodically in hominids [56]. In cichlids, *foxp2* is expressed in distinct foci in the thalamus and telencephalon, as well as in the pharyngeal arches where sound is produced [57], in the otic placodes and tectum where sound is received and processed, and in the fins.

### Hedgehog pathway

The Hedgehog pathway executes pervasive roles in embryonic development, stem cell renewal, and cancer biology. Hedgehog proteins are a group of soluble morphogens that include Indian Hedgehog, Desert Hedgehog, and perhaps the most well studied ligand in embryology, Sonic Hedgehog (SHH). These morphogens bind to the transmembrane receptor Patched (Ptch), releasing co-receptor Smoothened (Smo) and permitting activation of Hedgehog signaling. The Hh pathway is involved in the specification and morphogenesis of nearly all animal organs [58].

**Fig. 6:**
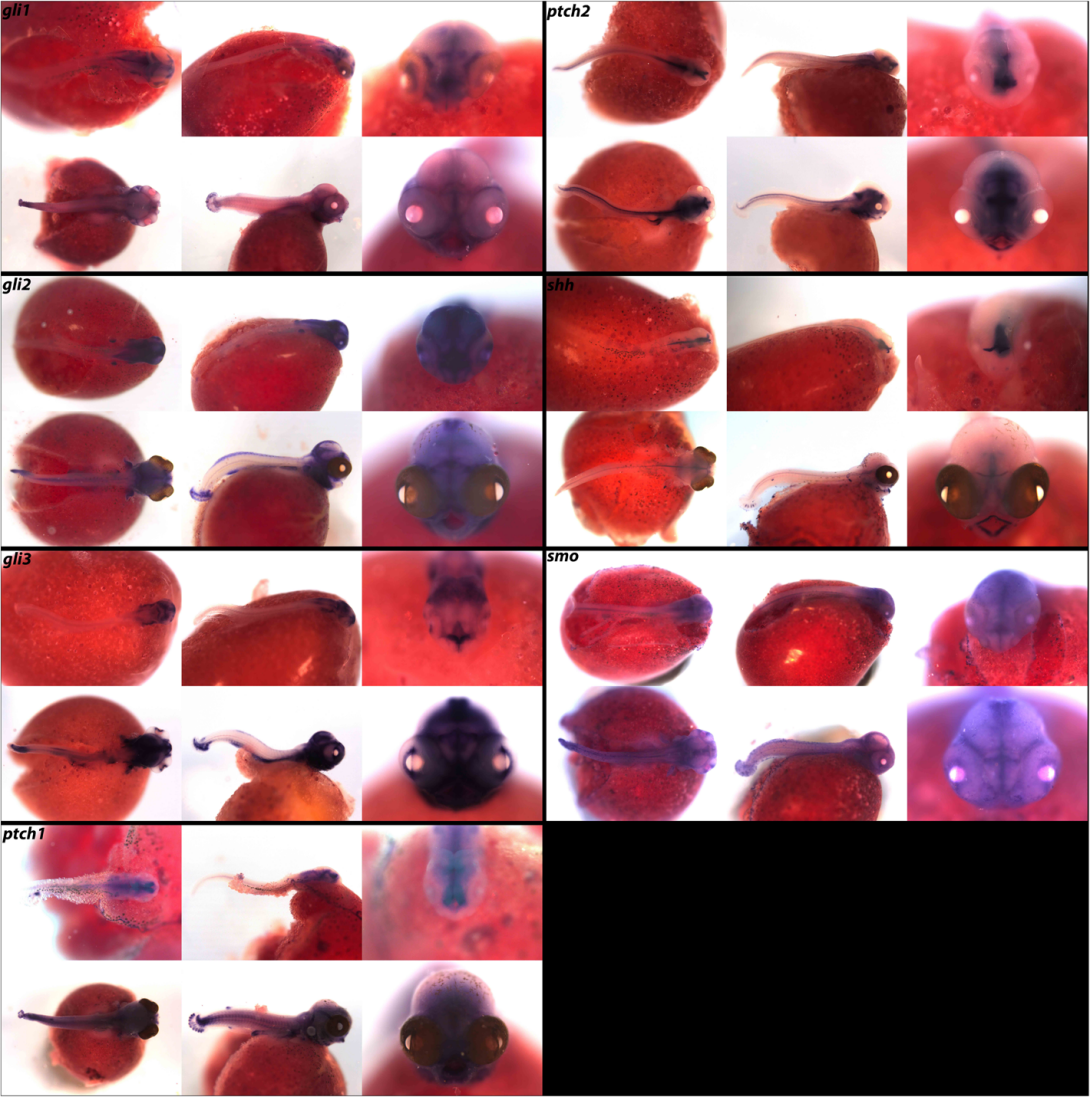
Expression of Hedgehog pathway genes at pharyngeal and larval stages. Imaged whole-mount in dorsal, lateral, and frontal orientations

In Fig. 6, we report expression of transcription factors *gli1*, *gli2* and *gli3*, receptors *ptch1*, *ptch2* and *smo*, and the ligand *shh*. Similar to expression seen in zebrafish [59], we find that all of the Hh pathway genes included exhibit t-shaped expression in the ZLI boundary of the diencephalon at both stages, as well as expression in the pharynx and fins (Table 4). We observe heavy expression of *gli2* and *gli3* in the midbrain and dorsal telencephalon, but expression of *gli1* is less prominent*.* In cichlids, *ptch1* is responsible for adaptive variation in jaw shape [60] and function [14]. We see *ptch1* and *ptch2* expressed in the jaw and throughout the central nervous system and somites, and *ptch1* additionally in the olfactory and otic cups. Ligand *shh* exhibits similar but more restricted patterns of expression in the forebrain, jaw, and somites. Similar to results found in zebrafish [61], *smo* appears at the midline and somites, as well as in the brain and fins at both stages.

### Homeobox pathway

**Fig. 7:**
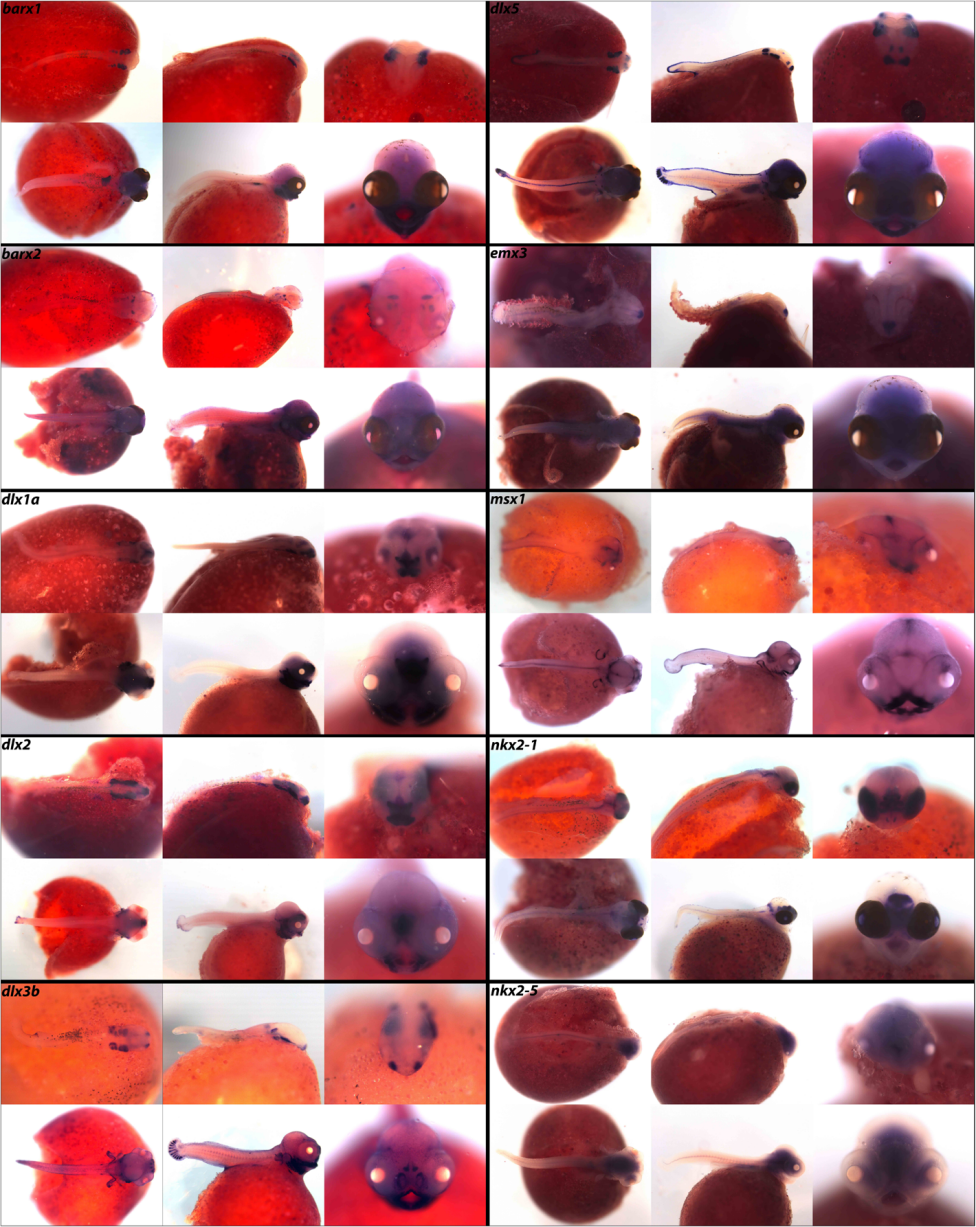
Expression of ANTP class Homeobox genes at pharyngeal and larval stages. Imaged whole-mount in dorsal, lateral, and frontal orientations

Called the “Rosetta Stone of developmental biology,” the homeobox gene family is best known for its role in organizing the metazoan body plan. Hallmark to this family is the “homeobox,” a conserved homeodomain sequence approximately 60 amino acids in length that binds DNA. With an estimated 300 homeobox genes, comprised of true genes and pseudogenes, Hox transcription factors are often divided into classes (approximately eleven) and subclasses that represent their general developmental functions [62]. Belonging to the *Antennapedia* gene of *Drosophila* (ANTP) class, we report expression in cichlids of *barx1* and *barx2* of the NK-like (NKL) subclass (Fig. 7). *barx1* expression has previously been demonstrated in cichlid pharyngeal teeth [63], and here we describe expression of *barx1* and *2* in the oral jaws, pharynx, and the gut.

*dlx1a*, *dlx2*, *dlx3b*, *dlx5, emx3*, *msx1*, *nkx2-1* and *nkx2-5* belong to the ANTP class and members have been well described as mediators of zebrafish jaw [64] and lamprey pharynx development [65], as well as important for fish brain development [66]. In Fig. 7 we note strikingly similar expression of *dlx1a* and *dlx2*, and expression of Dlx and Msx genes in the jaws and pharynx. The Dlx and Nkx genes are expressed in the ventral regions of the mid and/or forebrain. *emx3* is expressed in the dorsal telencephalon early, and at later stages is seen throughout the brain and trunk.

**Fig. 8:**
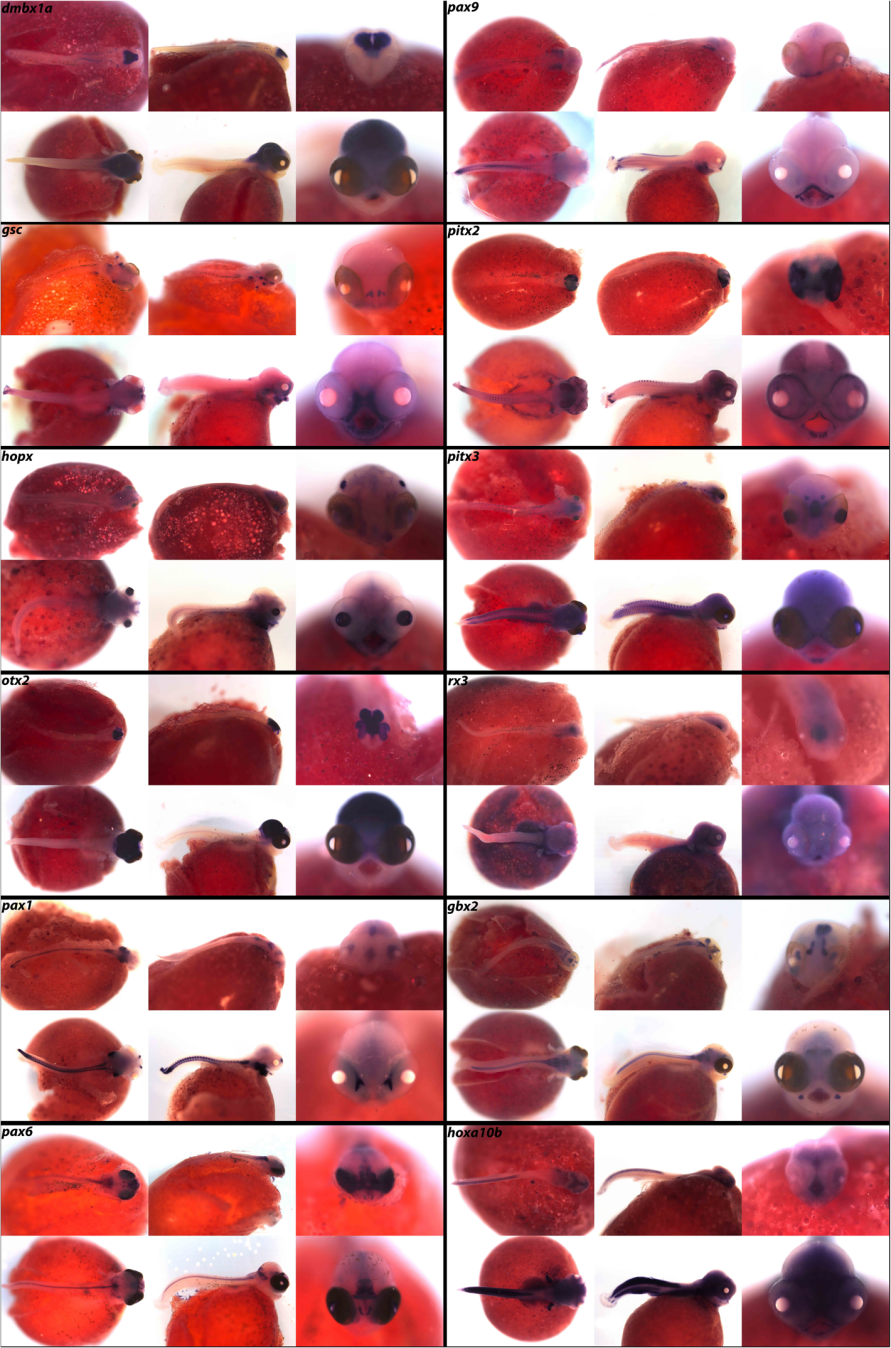
Expression of PRD and HOXL Homeobox genes at pharyngeal and larval stages. Imaged whole-mount in dorsal, lateral, and frontal orientations

We also present expression of *Paired* gene of *Drosophila* PRD class factors *dmbx1a, gsc*, *hopx*, *otx2*, *pax1, pax6, pax9*, *pitx2, pitx3* and *rx3*. Many of the members of the PRD class are known to be important for eye development [67–69], and we note expression of each of these factors in either retinal or lens development at the pharyngula or larval stages. *dmbx1a* exhibits expression in the midbrain at 4dpf, and at 6dpf expression is visible throughout the optic tectum and hindbrain. *gsc* and *hopx* are both expressed in the pharynx and eye structures, while *otx2* exhibits heavy expression in the fore-, midbrain and eyes. In Fig. 8 we note expression of the Pax genes in the somites, and expression of *pax1* and *pax9* in the pharynx and jaw. Paired-Like Homeodomain factors *pitx2* and *pitx3* demonstrate expression in the eyes, brains, and somites, while *rx3* is localized to the presumptive hypothalamus/preoptic region.

*gbx2* and *hoxa10b* of the HOXL subclass are expressed in foci of the jaw joint [70] and fins respectively, the latter of which has been described as crucial for proper limb and fin patterning [71].

In the three amino acid loop extension (TALE) superclass we demonstrate expression of *irx1b*, *irx2* and *meis2,* all three of which are expressed in the eyes and brain. Belonging to the LIM class we present expression of LIM homeobox 2 (*lhx2), lhx6* and *lhx9. lhx2* and *lhx9* exhibit essentially identical expression patterns in the brain, fins, and spinal region, while *lhx6* is only expressed in the jaw, pharynx, and preoptic region.

**Fig. 9:**
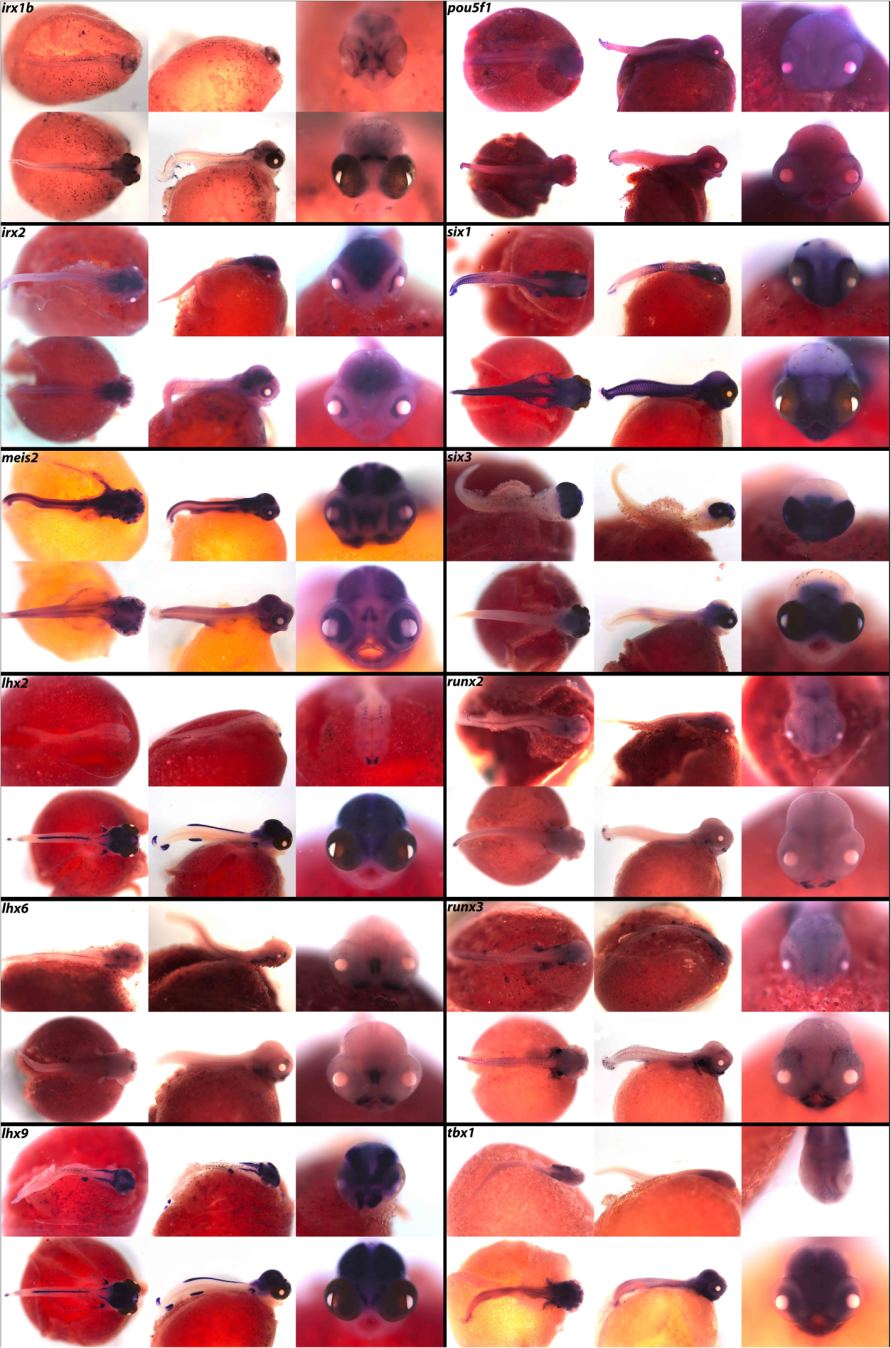
Expression of Homeobox genes at pharyngeal and larval stages. Imaged whole-mount in dorsal, lateral, and frontal orientations

In the POU class, named for Pit, Oct, and Unc transcription factors (POU class), we document expression of *pou5f1,* and in the SINE class we show expression of *six1* and *six3*. In Fig. 9 we observe expression of *six1* in the brain, eyes, somites, and fins, while *six3* is expressed only in the diencephalon, telencephalon, nasal placodes and eyes. Finally, we report expression of *runx2* and *runx3* in the jaws and pharynx, and transcription factor *tbx1* throughout the brain and in the fins.

### Calcium, endocrine, and insulin signaling

**Fig. 10:**
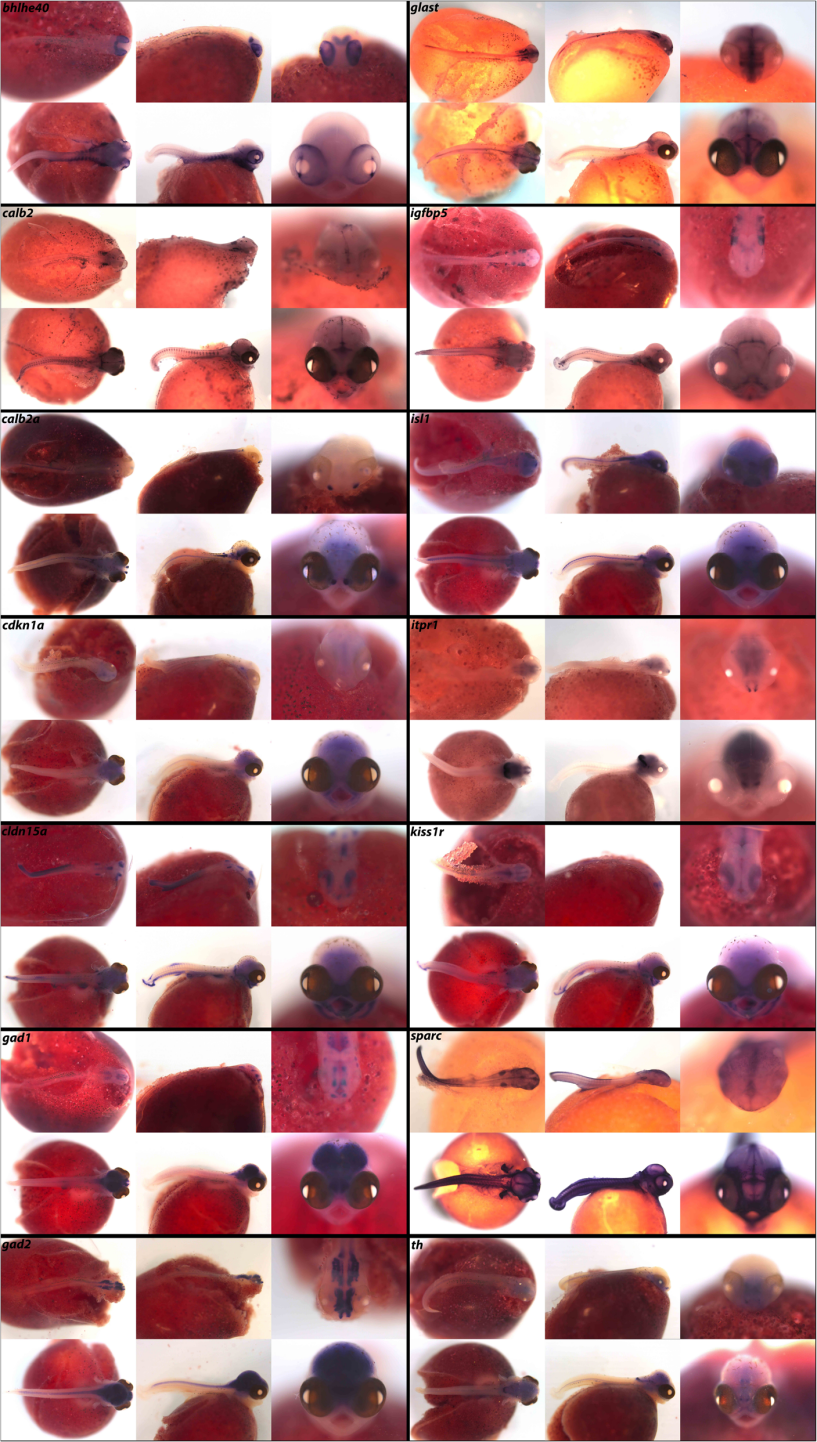
Expression of Calcium, Endocrine, and Insulin signaling factors at pharyngeal and larval stages. Imaged whole-mount in dorsal, lateral, and frontal orientation

While proteins such as insulin and regulators of calcium are essential for maintaining endocrine homeostasis in adult animals, their roles in embryogenesis are pervasive, but not well known. Entire families of signaling proteins exist to precisely coordinate cellular communication and resulting developmental differentiation, often in the same places where they will signal later in ontogeny. One example is calcium signaling, which is essential for proper odorant detection and olfaction [72]. In Fig. 10, we note expression of calcium signaling regulators *calb2*, *calb2a*, *cldn15a*, *kiss1r*, and *sparc* in the olfactory placodes and resulting nasal epithelium. We also observe expression of *calb2* in the cephalic lateral line, pharynx, gut, and somites, and *calb2a* in the hindbrain, as well as in the spinal region. Tight junction factor *cldn15a* is expressed in the three brain regions, pharynx, fins and jaw, while *itpr1* expression is restricted to the forebrain and cerebellum (Table 6). Receptor *kiss1r* exhibits notable expression in the eyes and hindbrain at 4dpf, and by 6dpf expression also appears in the jaw and pharynx. Extracellular matrix factor *sparc* is expressed generally across the integument and along the cephalic lateral line and spinal region.

As another example, *gad1* and *gad2* (also known as *gad67* and *gad65*, respectively) encode enzymes for the production of the neurotransmitter GABA and have known roles in schizophrenia and Parkinson’s disease. As in zebrafish [73] and other organisms, we note expression of *gad1* and *gad2* throughout the brain early in pharyngeal and larval stages of development. Plasma membrane transporter *glast* is expressed in cephalic lateral line placodes, where ion exchange will mediate sensory signaling later in the fully functional organ. In the insulin pathway, we report expression of *igfbp5* in lateral line and all brachial arches. *isl1*, or Insulin gene enhancer protein, binds to insulin enhancer sites to regulate insulin gene expression and has known roles in diabetic disease. It is commonly used as a marker of pancreatic cells early and late in zebrafish ontogeny [74] and we show is expressed similarly in cichlids, as well as in forebrain neuronal subsets. Factors involved in endocrine signaling, such as *bhlhe40, cdkn1a,* and *th,* are all diffusely expressed in the brain. *bhlhe40* exhibits notable expression in the eyes at both stages, and additional expression in the pharynx and somites at 6dpf.

### Mitogens, stem cell factors and tumor suppressors

**Fig. 11:**
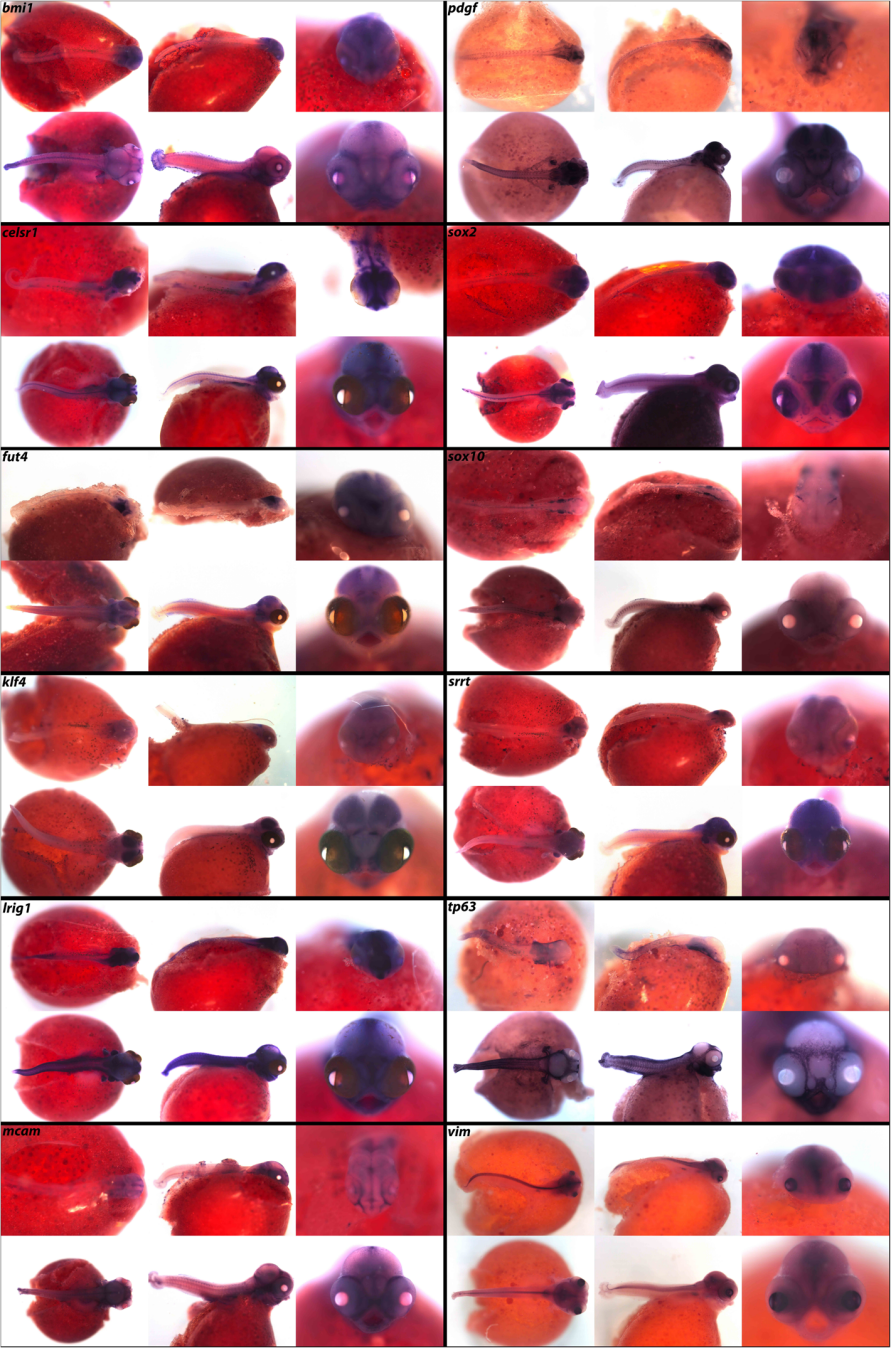
Expression of WNT pathway genes at pharyngeal and larval stages. Imaged whole-mount in dorsal, lateral, and frontal orientations

The biomedical world has greatly invested in understanding the processes of cellular renewal and division because of implications in regenerative medicine and cancer. Despite this focus, little attention has been paid to embryo-wide expression patterns of the genes involved (Fig. 11). *bmi1,* an epithelial stem cell marker in intestinal tissues, along with *lgr5* [75], is expressed along the lateral line and in the brain, eyes and fins (see Fig. 11 for expression of *lgr4* and *lgr6*). *fut4* is a reported mitogenic factor involved in tumor suppression [76] expressed in cichlid brain structures. Arsenate resistance protein, *srrt*, has been shown to promote self-renewal of mouse neural stem cells by regulating *sox2* expression [77]. We observe expression of *srrt* in the hindbrain and eyes.

Mesenchymal stem cells (mSCs) can be difficult to define due their loose spatial arrangement and degrees of potency. We report expression of *celsr1*, *mcam, pdgf*, and *vim*, which have recently been hypothesized to maintain mSCs [78, 79] in structures including brain, eyes, lateral line, and fins.

Crucial to the dichotomy of stem cell potency is the genetic environment that houses these cells, known as the niche. For instance, a set of key genes known as the Yamanaka factors, *cmyc*, *klf4*, *oct4*, and *sox2* are important for maintaining pluripotent stem cells (PSCs), and through retroviral induction can transfate mouse fibroblasts into induced PSCs (iPSCs) [80]. We have cloned *oct4*, reported as *pou5f1* in the Hox panel (Fig. 9). Similar to reports in zebrafish [81] we see little whole-mount expression of *oct4* past neurulation, presumably because of its defined roles in PSC maintenance. However, we report expression of *klf4*, noted in lateral line, fins, and brain, as well as *sox2*, noted even at later larval stages in adult organs capable of self-renewal, including teeth, taste buds, and the cephalic lateral line. The ability of *sox2* to persist and localize to epithelial stem cell (eSC) niches has been noted before [82]. *sox2* has been reported as an eSC marker in a host of adult organs [83, 84], along with *bmi1* and *lgr5* [75] in the intestine, and tumor suppressor *lrig1* as a master regulator of eSCs [85]. We observe *lrig1* throughout the brain, spinal region, fins, and eyes. Finally, we report expression of neural crest stem factor *sox10* in the pharynx and somites, and mitogenic factor *tp63* in the jaw, pharynx, CNS, cephalic lateral line, and fins.

### Notch pathway

The intercellular Notch signaling cascade is a highly conserved pathway involved in animal cell specification and proliferation [86]. Notch signaling exhibits versatility through a gamut of posttranslational modifications that alter receptor response to ligand. Notch activation occurs primarily by juxtacrine signaling from Delta, Serrate, and Jagged class ligands, which bind the Notch receptor extracellular domain of an adjacent cell. This binding causes proteolytic cleavage of a cytosolic domain to enable it to act as a transcription factor. The Notch pathway is of particular interest in axial patterning during embryogenesis because of this characteristic signal transduction between neighboring cells. In vertebrate models, including chicken [87], and mouse [88], temporal regulation of Notch in the pre-somitic mesoderm plays an important role in segmentation. In zebrafish, segmentation can be restored in Notch-deficient embryos via delivery of artificial pulses of Notch [89]. In cichlids, the Notch pathway is involved in patterning and regeneration of teeth [32], and in the renewing mouse incisor Notch has a role in maintaining the stem niche [90].

**Fig. 12:**
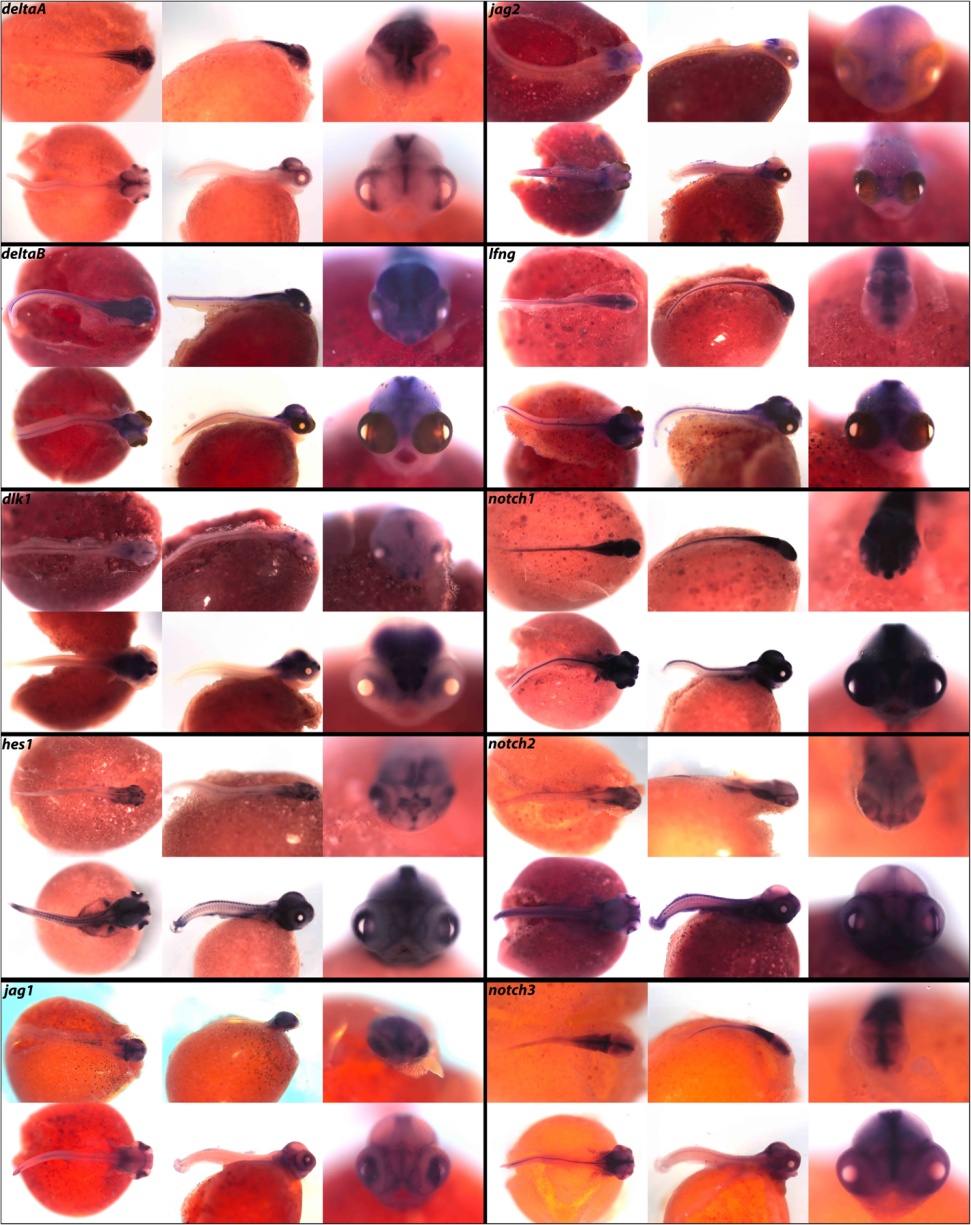
Expression of Notch pathway genes at pharyngeal and larval stages. Imaged whole-mount in dorsal, lateral, and frontal orientations

In Fig. 12 we document expression of *deltaA*, *deltaB*, *dlk1, jag1*, and *jag2* ligands, Notch inhibitor *lnfg*, transcription factor *hes1,* and *notch1*, *notch2*, and *notch3* receptors. As indicated in Table 8, we observe *deltaA* throughout the brain at 4dpf, with notable expression in the mid- and hindbrain. By 6dpf, expression is concentrated along the central midline of the fore- and midbrain and around the eyes. *deltaB* exhibits a similar pattern in the brain, but has additional expression in the somites and pharynx at both stages.

In Drosophila, Notch signaling has been shown to regulate cell fate in the eye by acting at specific ommatidium photoreceptors [91]. We observe expression in the retina (see frontal view) for *deltaA, hes1, jag1, notch1* and *notch2,* as well as expression of *deltaB, dlk1*, *jag2,* and *notch3* in the eyes. At 4dpf, *dlk1* expression is restricted to small areas in all three regions of the brain and dorsal side of the eyes, and by 6dpf this expression has spread to the telencephalon, optic tectum, and hindbrain. *hes1* expression at 4dpf is seen in the head and lateral line, and at 6dpf expression is also evident in the vertebral somites and jaw.

Jagged1 is important for endothelial tissue development, and has been correlated with human congenital diseases of the heart [92]. We observe expression of *jag1* and *jag2* throughout the brain and fins at both developmental stages, and *jag2* additionally in the somites and jaw. *lnfg* is expressed in the brain and eyes, with heavy expression along the central midline. We also observe *lnfg* in the somites, where it is critical for somite segmentation according to studies performed in mouse [93]. Notch receptors *notch1*, *notch2* and *notch3* all exhibit expression along the center midline of the brain and in the jaw, somites, pharynx, and lateral line.

### Brain development and neurogenesis

The formation of the brain and nervous system is highly conserved and requires the integration of many, often competing, molecular signals. Cichlid brains evolve diversity via subtle modification of conserved gene regulatory networks [6, 19]. Here we show expression of transcription factors and other components of nervous system development as well as guidance cues involved in neurogenesis and axonal growth.

**Fig. 13:**
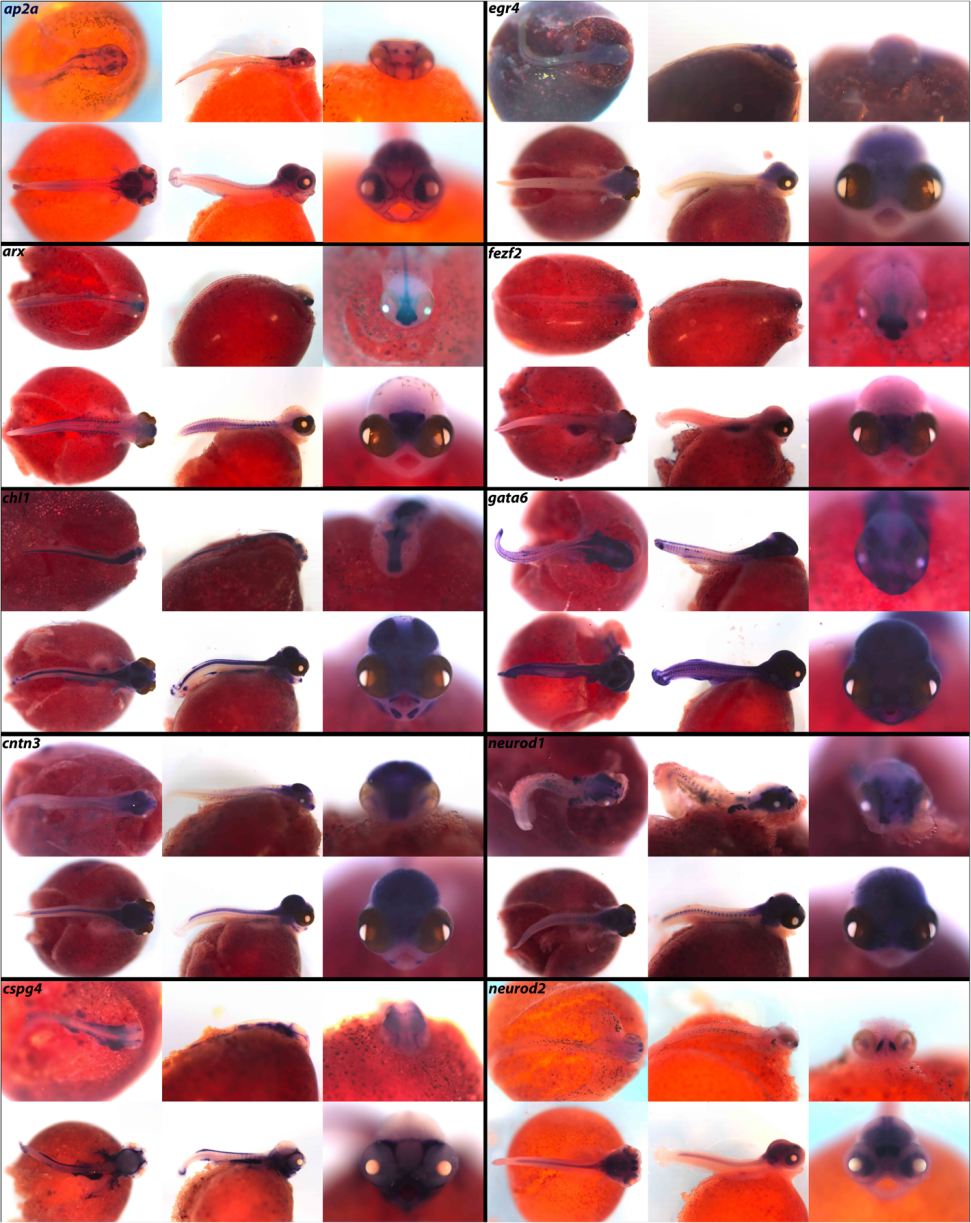
Expression of brain development and neurogenesis factors at pharyngeal and larval stages. Imaged whole-mount in dorsal, lateral, and frontal orientations

In vertebrates, transcription factor *ap2a* is required for ectodermal migration during neural tube closure and cell fate specification, and mediates regulatory networks that drive neural crest evolution [94]. In Fig. 13, *ap2a* is notably expressed in the eyes and brain. We observe *arx,* mutations of which are linked to improper CNS formation and mental retardation [95], in the somites, spinal region, and in a triangular pattern in the forebrain. Neural adhesion molecule gene *chl1* is heavily expressed throughout the CNS, jaws, fins, and lateral line. We observe *cntn3,* a promoter of neurite outgrowth, throughout the CNS, in the eye, and in the jaw joint. Integral membrane proteoglycan *cspg4* is expressed in the pharynx, gut, and dorsal fins. *fezf2* expression in the telencephalon exhibits a triangular pattern similar to that of *arx.* We observe expression of transcription factor *tbr1* in the telencephalon, olfactory bulbs and eyes, similar to the results reported in zebrafish [48]. Glutamate transporter *vglut2.1* expression is only weakly in the eyes and throughout the brain. *zash1,* involved in body segment formation and Hox regulation [96], is seen in the brain and eyes. Disruption of highly conserved transcription factor *gata6* demonstrates its role in vertebrate development [97]. We observe *gata6* heavily expressed throughout the brain, somites, fins, and gut.

In Fig. 13, *egr4* is expressed throughout the brain at 6dpf while neuronal differentiation factor *neurod1* can be seen in the brain and pharyngeal arches. *neurod2* expression at 4dpf is localized to the telencephalon and eyes, but at 6dpf this expression appears throughout the brain and nerve cord. *neurog1* expression is evident in the brain and the dorsal trunk.

**Fig. 14:**
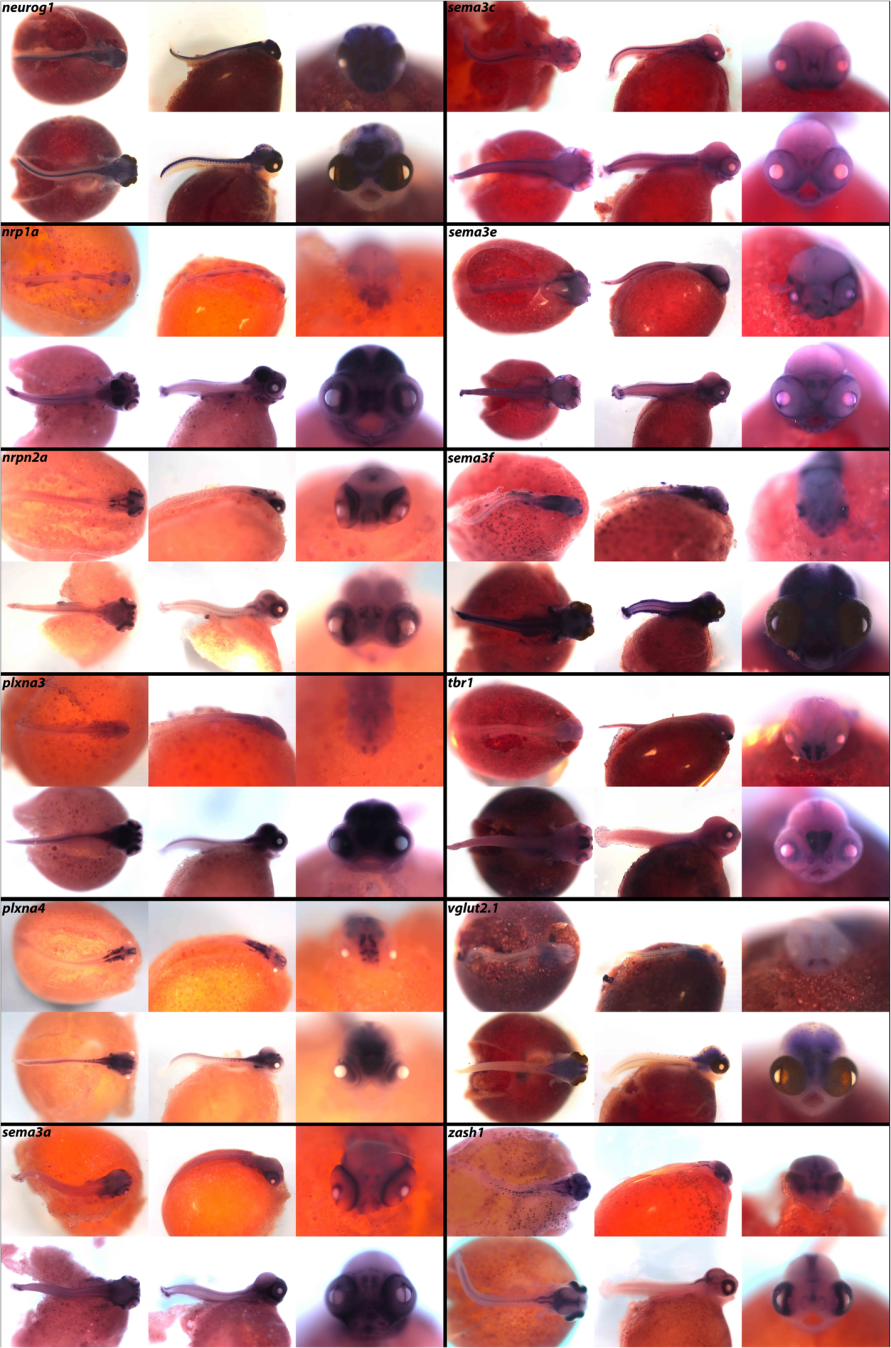
Expression of brain development and neurogenesis factors at pharyngeal and larval stages. Imaged whole-mount in dorsal, lateral, and frontal orientations

Semaphorins are a family of secreted and membrane-bound proteins that guide the axonal growth cone during neurogenesis [98]. The semaphorin superfamily is divided into eight subclasses, all of which have a conserved 500 amino acid N-terminal sema domain [98] with variable C-terminals. In Fig. 14 we show expression of class 3 semaphorins, present in vertebrates, which are secreted proteins that act through a heterocomplex receptor of transmembrane plexins, cell adhesion molecules, and neuropilins. Specific combinations of these three receptor components allow selective binding of different Semaphorin 3 genes depending on cell type, developmental stage, and location. A model developed in the mouse molar indicates that the Wnt and Tgf-β pathways signal from the dental epithelium to *sema3a* in the adjacent mesenchyme, which acts to guide the growing axon via short range repulsion along the boundaries of the nerve pathway [99].

We observe receptors *nrp1a* and *nrpn2a* in similar patterns in the eyes and brain, with heavier expression of *nrp1a* in the fore/midbrain and pharynx. *plxna3* is expressed throughout the brain and eyes, while *plxna4* is localized to more restricted regions of the eyes and in the dorsal region of the cerebellum. We report expression of *semaphorins 3a, 3c, 3e,* and *3f* in the retinal tissue similar to expression reported in zebrafish [100], at both the pharyngula and larval stages. All four of these *semaphorins* are expressed in the early jaw, pharynx, nasal pits, somites, and presumptive optic and otic regions.

### Wingless pathway

The Wingless (Wnt) signaling pathway involves many factors that alter transcription, regulate calcium levels, and affect cell polarity during embryonic development through paracrine and autocrine signal transduction. Wnt ligands initiate the pathway by binding the N-terminal extracellular domain of Frizzled family receptors, which then bind cytoplasmic Dishevelled within the cell to propagate the signal. This pathway is highly conserved across vertebrates and invertebrates, with more than 20 mammalian Wnt ligands identified [101].

**Fig. 15:**
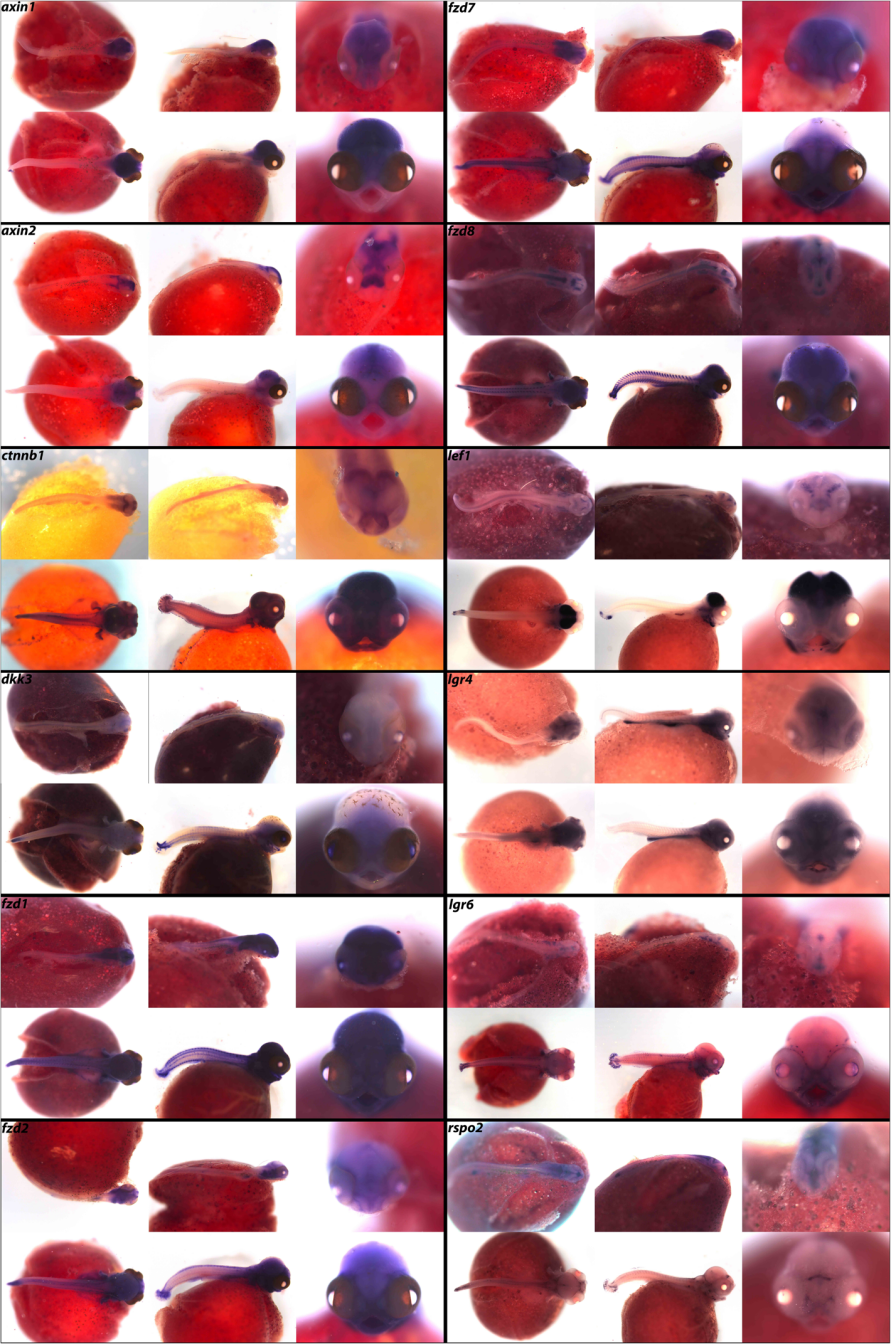
Expression of WNT pathway genes at pharyngeal and larval stages. Imaged whole-mount in dorsal, lateral, and frontal orientations

The canonical Wnt pathway regulates transcription by the translocation of cytoplasmic β-catenin (*ctnnb1*) into the nucleus, where it co-activates Tcf/Lef family transcription factors. In the absence of Wnt activation, cytoplasmic β-catenin is ubiquitinated for proteasomal destruction by a complex containing Axin, APC, and GSK3 proteins. In Fig. 15, we observe expression of *axin1*, *axin2* and *ctnnb1* in the brain, and additional *ctnnb1* expression along the cichlid trunk, pharynx, jaw, and fins. Dickkopf family inhibitor *dkk3* exhibits expression in the brain, eyes, pharynx, and vertebral region. We also include four Frizzled family receptors, *fzd1*, *fzd2*, *fzd7 and fzd8,* which demonstrate similar expression patterns in the brain, somites, fins, jaw, and pharynx.

Developmental roles for Wnt signaling have been demonstrated for decades, and knowledge of the effects of this pathway has continued to grow. In 1980, lethal mutations of *wingless* were shown to affect *Drosophila* larvae body segments, making boundaries between body axes indistinguishable [102]. This was further demonstrated in *Xenopus* embryos, which exhibited duplicated axes when injected with mouse *Wnt1* RNA [103], and similar duplication was observed by injection of other Wnt related factors. This pathway is important for regulation of cell fate in self-renewing tissues, including mouse intestinal epithelium [104], and in zebrafish has been shown to be important in early neural crest development.

Nuclear β-catenin mediates transcriptional activation by transcription factors Lef1 and Tcf*.* We observe notable expression of *lef1* in the midbrain and forebrain in a similar pattern to that of *tcf712* in Fig. 15. *tcf3* exhibits expression in the brain, eyes, fins, and somites. Additionally, we report R-spondin receptors *lgr4* and *lgr6* in distinct patterns in the brain, gut, eyes, fins, and somites, and the secreted R-spondin *rspo2* in restricted regions of the fore- and hindbrain.

**Fig. 16:**
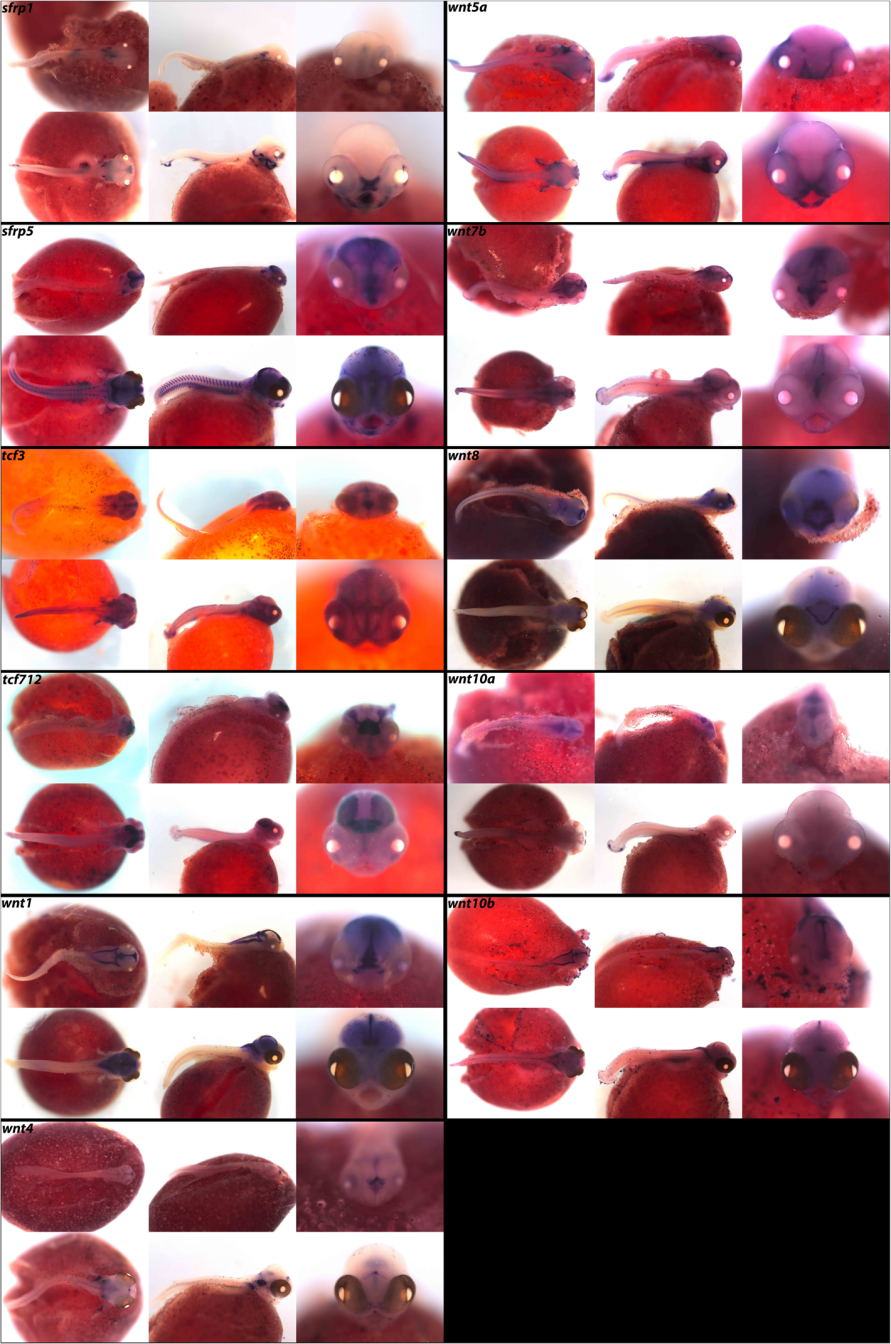
Expression of developmental genes at pharyngeal and larval stages. Imaged whole-mount in dorsal, lateral, and frontal orientations

In Fig. 16, we show expression of Wnt antagonist *sfrp1* in the hindbrain at 4dpf, and at 6dpf in the pharynx, eyes, jaw, and olfactory bulbs. *sfrp5* is expressed in all three brain regions, somites, jaw, and pharynx (Table 10). In cichlids, Wnt signaling is thought to affect bone deposition to regulate phenotypic changes in craniofacial development [15]. *wnt1* and *wnt8* are involved in telencephalon and diencephalon development [6]. We see secreted Wnt ligands *wnt1*, *wnt4*, *wnt5a*, *wnt7b*, *wnt8*, *wnt10a* and *wnt10b* expressed in the hindbrain, fins and pharynx and specific Wnts, for example *wnt10b* and *wnt5a,* differentially expressed in the midbrain.

### Other developmentally expressed genes

**Fig. 17:**
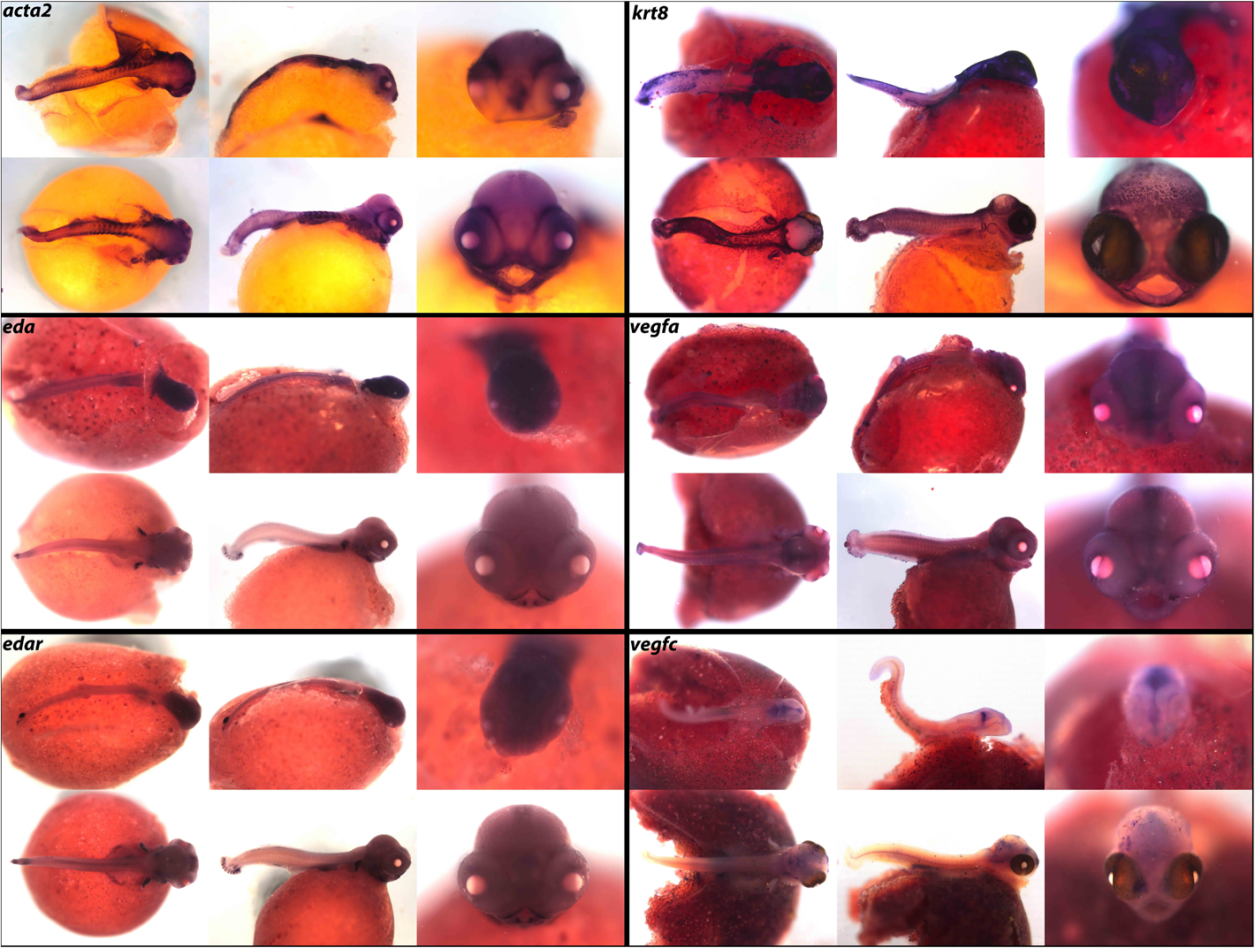
Expression of Mitogens, Stem, and Tumor Suppressor factors at pharyngeal and larval stages. Imaged whole-mount in dorsal, lateral, and frontal orientations

Our final expression panel (Fig. 17) includes factors not specifically involved in the above pathways and processes. We show factors involved in muscle contraction including actin gene *acta2* and keratin *krt8*, which polymerizes into cytoplasmic filaments in epithelial cells. As in zebrafish, smooth muscle actin *acta2* is expressed in the myotomes of the trunk and in the intestinal musculature [105], and we also observe expression around the eyes.

Transmembrane protein ectodysplasin A (*eda*) acts through receptor *edar* in ectodermal tissue development. This signaling pair helps pattern early embryonic structures including skin, hair, and teeth, from germ layers, and outlines placode derived structures such as scales and precisely patterned chicken feathers [106]. We observe *eda* and *edar* localized to the tooth placodes and fins at these stages, and note expression in and around scales later in development (not shown). Both factors appear to be expressed more heavily in pharyngula stage than at the larval stage. We see *krt8* expressed generally across the entire integument at both stages of development.

Vascular endothelial growth factors *vegfa* and *vegfc* are involved in angiogenesis, vasculogenesis, and cell migration. Overexpression of this family of genes is seen in the vascularization of cancerous tumors [107], and is the target of many emerging cancer therapies. We observe expression of *vegfa* along the midline of the brain, as well as in the hindbrain and somites. We also observe *vegfc* in the somites, as well as in the olfactory, optic, and otic regions.

## Conclusions

### Novel expression domains

Here, we provide a set of probes for spatial analysis of gene expression, useful across hundreds of East African cichlid fishes, for studies of evolution and development. Gene expression patterns are captivating, and provide important clues to the evolution of gene regulation. Gene expression is context-dependent, dynamic in space and time. Our compendium of gene expression for early Lake Malawi cichlid development provides examples of (i) expression patterns conserved with many other animals, as well as (ii) expression patterns that can be considered novel, because they haven’t been assayed at these particular spaces and times. We highlight a few of these novel expression domains.

Calcium and endocrine signals (Fig. 10) as well as the stem cell/mitogenic factors (Fig. 17) are rarely studied at these stages, in whole mount. Particularly striking spatially delimited gene expression patterns are observed for many of these genes, including *calb2*, *calb2a*, *cldn15a*, *kiss1r*, *glast*, *sparc*, *stra13*, *bmi1*, *pdgf*, *celsr1*, *klf4*, *trp63* and *vim*, suggestive of precise roles in embryonic development. We also observe new expression domains from well-studied genes. Notable from this class are *osr2* (Fig. 3; expression in fins) *foxp2* (Fig. 5; expression in fins and jaws), *hopx* (Fig. 7; expression in the pharynx), *nrp1a*, *sema3a*, *sema3c* and *sema3e* (Fig. 14; expression in fins and jaws). These novel expression domains set the stage for future exploration of function.

## Abbreviations

BMP: Bone morphogenetic pathway
FGF: Fibroblast growth factor
FOX: Forkhead box
Hh: Hedgehog
HOX: Homeobox
ISH: In-situ hybridization
*LF*: *Labeotropheus fuelleborni*
*MZ*: *Metriaclima zebra*
QTL: Quantitative trait loci
TGF: Transforming growth factor
Wnt: Wingless
